# Conformational Study of an Artificial Metal-Dependent Regulation Site for Use in Designer Proteins

**DOI:** 10.1002/zaac.201300131

**Published:** 2013-05-21

**Authors:** Emmanuel Oheix, Neil Spencer, Lee A Gethings, Anna F A Peacock

**Affiliations:** a School of Chemistry, University of BirminghamEdgbaston, B15 2TT, UK; b Waters CorporationAtlas Park, Simonsway, Wythenshawe, Manchester, M22 5PP, UK

**Keywords:** Bioinorganic chemistry, Metalloswitches, Bipyridine, Terpyridine, Peptides

## Abstract

This report describes the dimerisation of glutathione, and by extension, other cysteine-containing peptides or protein fragments, with a 5, 5’-disubstituted-2, 2’-bipyridine or 6, 6”-disubstituted-2, 2’:6’,2”-terpyridine unit. The resulting **bipy**-**GS_2_** and **terpy**-**GS_2_** were investigated as potential metal ion dependent switches in aqueous solution, and were found to predominantly adopt the *transoïd* conformation at physiological pH. Metal complexation with Cu^II^ and Zn^II^ at this pH has been studied by UV/Vis, CD, NMR and ion-mobility mass spectrometry. Zn^II^ titrations are consistent with the formation of a 1:1 Zn^II^:**terpy**-**GS_2_** complex at pH 7.4, but **bipy**-**GS_2_** was shown to form both 1:1 and 1:2 complexes with the former being predominant under dilute micromolar conditions. Formation constants for the resulting 1:1 complexes were determined to be log *K_M_* 6.86 (**bipy**-**GS_2_**) and 6.22 (**terpy**-**GS_2_**), consistent with a higher affinity for the unconstrained bipyridine, compared to the strained terpyridine. Cu^II^ coordination involves the initial formation of 1:1 complexes, followed by 1.5Cu:1**bipy**-**GS_2_** and 2Cu:1**terpy**-**GS_2_** complexes at micromolar concentrations. Binding constants for formation of the 1:1 complexes (log *K_M_* 12.5 (**bipy**-**GS_2_**); 8.04 and 7.14 (**terpy**-**GS_2_**)) indicate a higher affinity for Cu^II^ than Zn^II^. Finally, ion-mobility MS studies detected the free ligands in their protonated form, and were consistent with the formation of two different Cu adducts with different conformations in the gas-phase. We illustrate that the bipyridine and terpyridine dimerisation units can behave like conformational switches in response to Cu/Zn complexation, and propose that in future these can be employed in synthetic biology with larger peptide or protein fragments, to control large scale folding and related biological function.

## Introduction

The primary, secondary, tertiary and quaternary structure of proteins, are all crucial in controlling biomolecular recognition events and biological function. In some cases small local structural changes can greatly impact the secondary, tertiary and even quaternary structure of a whole domain, and as a result can influence the biological activity or function. Regulation processes can arise from contact with other biomolecules, and can themselves sometimes be triggered in response to an external stimuli such as pH.^1^ Substructure stabilisation or destabilisation, in response to an external stimulus or binding of an effector molecule, allows the protein to communicate molecular events over long distances, through a large variety of signal-response pathways.^2^ Allostery is the generic term used to describe a regulation site which is distinct from the active site in a biomolecule.

Chemists have invested significant effort into trying to model or engineer allosteric sites.^3^ One attractive application is to introduce these into artificial biomolecular constructs for use in synthetic biology.^4^ Metal complexation by bipyridine (bipy) and terpyridine (terpy) have previously been exploited as allosteric switches in organic solvents,^5^,^6^ and have been inserted into a range of macromolecules including polymers^7^ and DNA.^8^ A number of reports describe their insertion into peptide sequences,^9^–^11^ however, the majority focus on using them to achieve dimerisation on metal ion complexation. Only a small fraction takes advantage of a *cisoïd-transoïd* conformational transition on metal ion complexation, in an effort to achieve allosteric regulation. Kelly and co-workers prepared several short peptides with a bipy unit introduced directly into the peptide backbone. The peptide sequence and the pH were found to influence the conformational state of the bipy, as a result the free ligand was only able to adopt the *transoïd* conformation under alkaline conditions. At pH = 9.5 they demonstrated that Cu^II^ coordination led to a structural reorganisation of the bipy linker to the *cisoïd* conformation, resulting in a secondary structure transition from random coil to β-sheet. However, the bpy linker could not be used as a switch at a physiologically relevant pH.^12^ This highlights the need for a better understanding of the factors (c.a. protonation of polypyridine, substituent nature and positioning) which govern the conformational state of bipyridine and related polypyridine units, if they are to be exploited as potential artificial allosteric regulation sites.

The aim of this work was therefore to study the potential for using these polypyridine units as conformational metal ion dependent switches in designed artificial protein architectures. We therefore prepared and studied two small model compounds comprising a polypyridine switchable unit substituted at either end with a short peptide (rather than a complex protein fragment). It was proposed that metal ion coordination would constitute the triggering event, resulting in a conformational change from *transoïd* to *cisoïd* and subsequent realignment of the two peptide moieties. Ultimately our goal is to extend this work to the alignment of larger peptides and small protein fragments, however, the preparation of model compounds containing short peptides (3 amino acids) will allow the conformational change of the polypyridyl linker to be studied in greater detail.

## Results

### Design and Synthesis

Various coupling techniques have been reported in the literature, however, in order for this approach to be widely applicable and for these polypyridyl units to be incorporated into structures based on natural protein motifs, we felt it was necessary to utilise native functionality for coupling. We therefore chose to take advantage of selective coupling to the sulfhydryl group of cysteine, the most nucleophilic naturally occurring amino acid. The model compounds reported here consist of polypyridyl linkers coupled through the Cys side chain of the naturally occurring tripeptide, glutathione (GSH).

**Figure 1:**
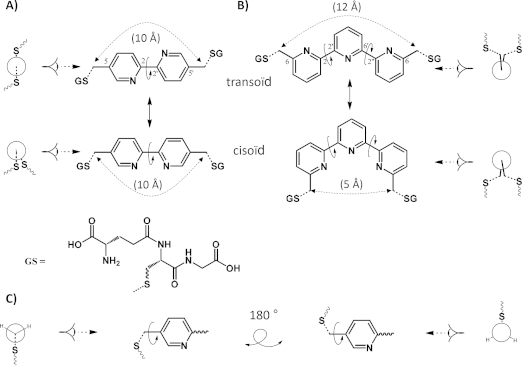
Schematic illustrating the impact of conformational transition in A) bipy-GS_2_ and B) terpy-GS_2_ on the inter-substituent distance and relative orientation. C) Illustration of the flexibility of the thioether linkage of (2-polypyridine) substituted at position 5-. Distances displayed are estimated based on reported structures for analogous compounds.^6^,^14^–^17^

Two different polypyridine units, differing in substitution pattern, were investigated. The conformation of the first, a 5, 5’-disubstituted bipyridine (bipy) model, was proposed to be largely unaffected by the presence of metal ions, as upon *transoïd* to *cisoïd* rearrangement, the substituent at position 5 of the 2, 2’-bipyridine experiences a 180° rotation around the 2, 2’-inter ring linkage, relative to the substituent at position 5’. As these two substituents are located along the rotation axis the distance separating them remains unchanged (see Figure 1A). In our design, one needs to further account for the length and the flexibility of the dimethyl sulfide bridge linking the polypyridyl unit and the peptide backbone. The dimethyl sulfide bridge can freely rotate (see Figure 1C), and is therefore able to effectively compensate for the rotation of the 2-pyridinyl. For these reasons 5, 5’-disubstituted-2, 2-bipyridine units can be considered allosteric ineffective.^13^

The second model compound contains a 6, 6”-disubstituted-2, 2’:6’,2”-terpyridine (terpy) as the linker unit. In contrast to the bipy linker, a *transoïd* to *cisoïd* rearrangement of the terpy linker would alter the relative distance between the two peptide substituents. Methyl-methyl distances for *cisoïd* and *transoïd* conformations of both model compounds, were estimated based on reported structural information for analogous compounds (see Figure 1).^6^,^14^–^17^

5, 5′-Dibromomethyl-2, 2′-bipyridine and 6, 6′′-dibromomethyl-2, 2′:6′,2′′-terpyridine were synthesized based on previously reported procedures,^18^ and were characterised by HR-MS, ^1^H and ^13^C NMR spectroscopy. The designed small model **bipy-GS_2_** and **terpy-GS_2_** allosteric regulation sites were synthesised by reacting each dibromomethyl linker with two equivalents of glutathione (GSH), in 50/50 mixture of acetonitrile and 100 mM aqueous Tris.HCl buffer pH 8.0. This method was adapted from previous reports.^9^ The model compounds, **bipy-GS_2_** and **terpy-GS_2_**, were purified by reversed phase C18-HPLC and fully characterised by ESI-MS, ^1^H, ^13^C NMR, UV/Vis spectroscopy and analytical HPLC (see Figure S1). The metal ion coordination chemistry and any associated conformational reorganisation of the model regulation sites, was studied by ^1^H NMR (Zn^II^ only), circular dichroism (CD), UV/Vis spectroscopy, and ion-mobility spectrometry (IMS) mass spectrometry (MS) over the millimolar to micromolar concentration range.

### Circular Dichroism (CD)

**Figure 2:**
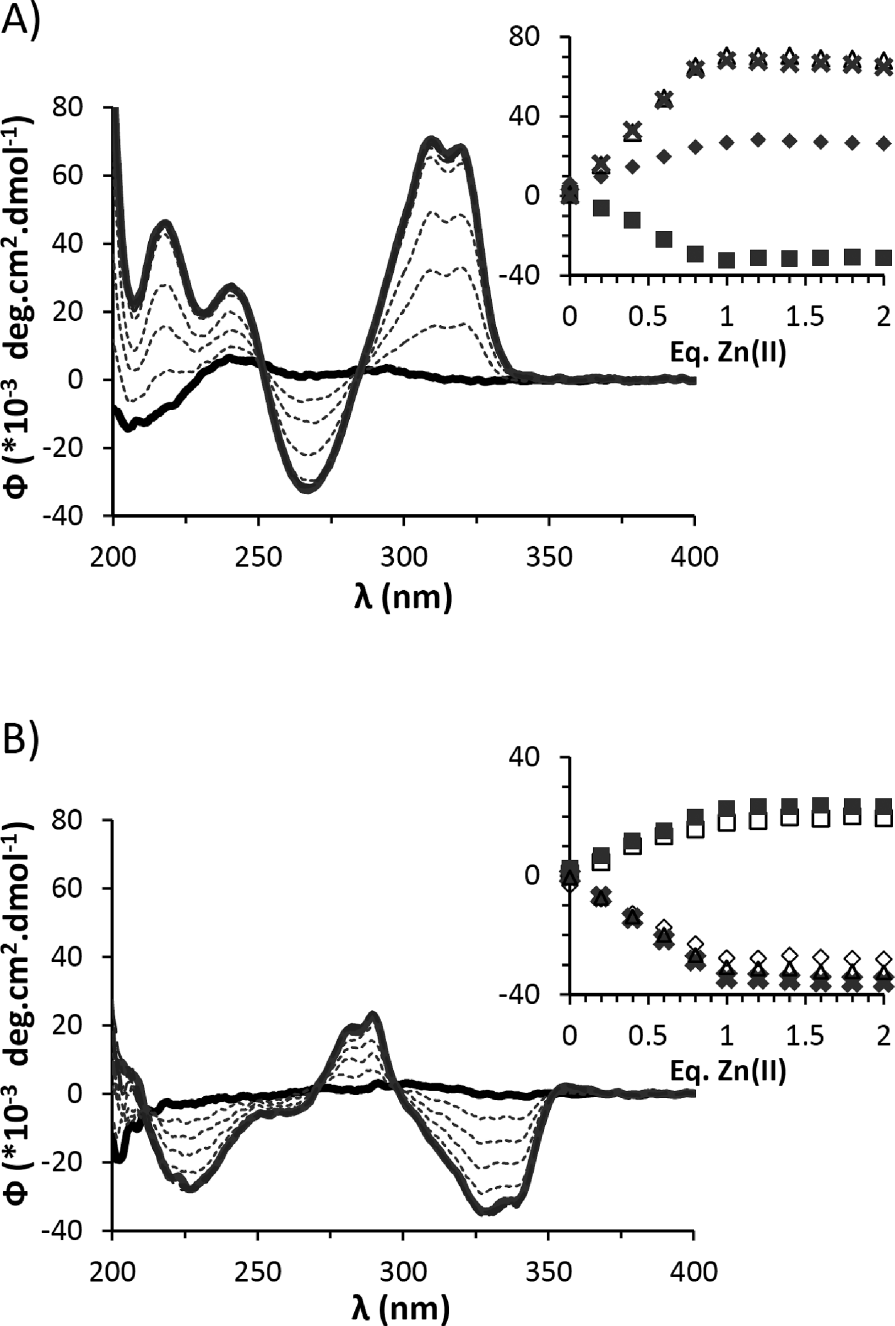
CD spectra for Zn^II^ titration of model switches. ZnCl_2_ titration into 350 μM of either (A) bipy-GS_2_ or (B) terpy-GS_2_ in 10 mM phosphate buffer pH 7.4. (black line) 0 equivalent, (dotted grey line) between 0 and 1 equivalent, (grey line) 1 equivalent, (half-dotted grey line) more than 1 equivalent ZnCl_2_. Inset of the molar ellipticity (A) 241 (♦), 266 (▪), 310 (Δ) and 320 nm (X); (B) 228 (◊), 283 (□), 290 (▪), 329 (X) and 340 nm (Δ) plotted as a function of the equivalence of Zn^II^.

The CD spectra of 350 μM solutions of **bipy-GS_2_** and **terpy-GS_2_**, recorded from 400 to 200 nm, did not display any notable signal. However, the addition of increasing concentrations of ZnCl_2_ to the solution of **bipy-GS_2_** at pH 7.4 led to the appearance of new positive transitions centred at 220, 241, 310 and 320 nm, as well as a negative transition at 266 nm, with isosbestic points at 251 and 285 nm (Figure 2A). In contrast, addition of ZnCl_2_ to **terpy-GS_2_** resulted in negative transitions at 228, 329, and 340 nm and positive transitions at 283 and 290 nm, with isosbestic points at 270 and 298 nm (Figure 2B). A plot of molar ellipticity as a function of ZnCl_2_ concentration reaches a plateau at ca. 0.9 (**bipy-GS_2_**) and 1.0 equivalents (**terpy-GS_2_**) of Zn^II^ per model switch (Figure 2).

**Figure 3:**
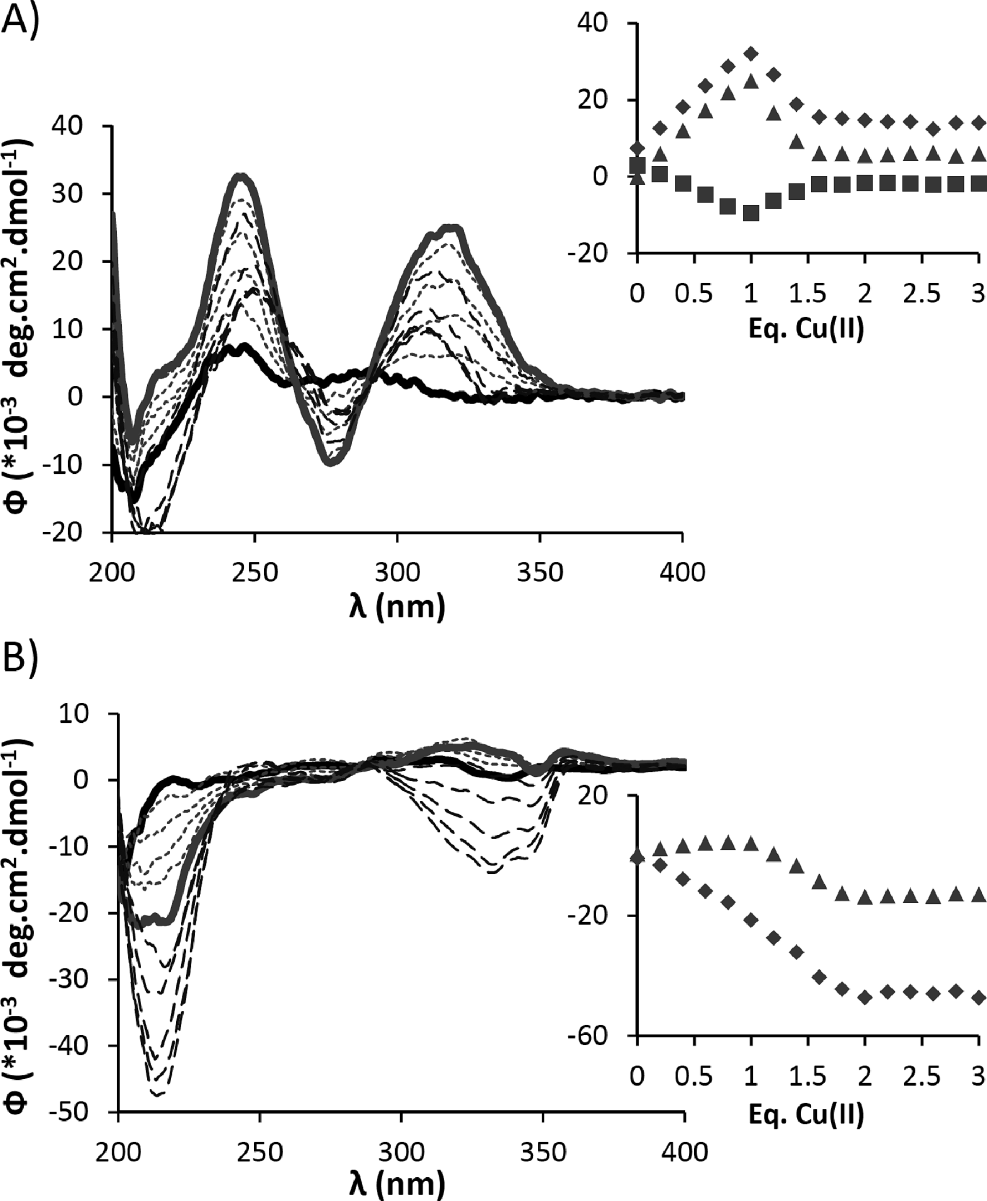
CD spectra for Cu^II^ titration of model switches. CuCl_2_ titration into 350 μM of either (A) bipy-GS_2_ or (B) terpy-GS_2_ in 10 mM phosphate buffer pH 7.4. (black line) 0 equivalent, (dotted line) between 0 and 1 equivalent, (grey line) 1 equivalent, (half-dotted line) between 1 and 2 equivalent CuCl_2_. Inset of the molar ellipticity (A) 247 (♦), 278 (▪) and 320 nm (▴); (B) 215 (♦) and 334 nm (▪)) plotted as a function of the equivalence of Cu^II^.

Similarly, the addition of CuCl_2_ to **bipy-GS_2_** (see Figure 3A) resulted in the appearance of positive transitions at 247 and 320 nm, and a negative transition centred at 278 nm. The peak intensities increase up to 1.0 equivalent of Cu^II^, with clear isosbestic points at 265 and 292 nm. Further addition of CuCl_2_ resulted in a gradual shift of the positive transitions toward 251 and 310 nm, and the negative transition toward 282 nm. All signals decreased in intensity, reaching a minimum on addition of 1.5 equivalents of CuCl_2_. No further spectral changes occur on addition of up to 3.0 equivalents CuCl_2_ (see Figure 3A) The titration of increasing concentrations of CuCl_2_ into a 350 μM solution of **terpy-GS_2_** at pH 7.4 resulted in the appearance of a negative transition centred at 215 nm. Two overlapping negative transitions at 335 and 347 nm appear on addition of more than 1.0 equivalent of CuCl_2_. A plot of molar ellipticity as a function of CuCl_2_ concentration reaches a plateau for all transitions at ca. 2.0 equivalents of Cu^II^ per **terpy-GS_2_** (see Figure 3B).

In all four cases, addition of excess EDTA (20 equiv. with respect to metal) resulted in CD spectra which were in good agreement with those of **bipy-GS_2_** and **terpy-GS_2_** recorded in the absence of metal ions.

### UV/Vis Spectroscopy

**Figure 4:**
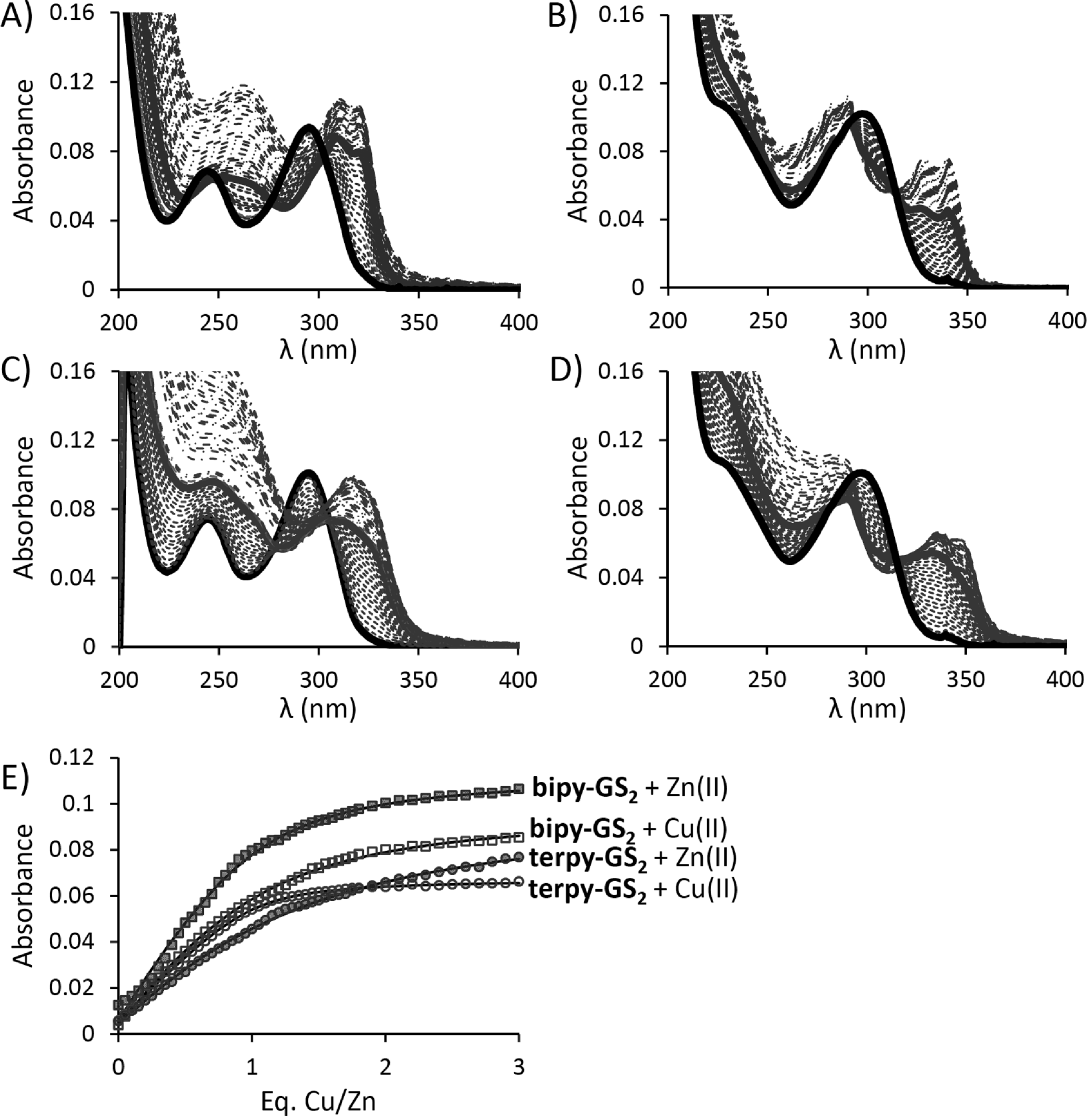
UV spectra for the metal titration of model switches. (A and B) ZnCl_2_ or (C and D) CuCl_2_ was added to solutions containing 5 μM of either bipy-GS_2_ (A and C) or terpy-GS_2_ (B and D) in 20 mM phosphate buffer pH 7.4. (black line) 0 equivalent metal added, (dark grey line) 1 equivalent CuCl_2_ added, (light grey line) 1 equivalent ZnCl_2_ added, (dark grey dotted line) or (light grey dotted line) between 0 and 1 equivalent of CuCl_2_ or ZnCl_2_ added, (dark grey half dotted line) or (light grey half dotted line) more than 1 equivalent added. For (C) buffer concentration was 100 mM and 20 mM glycine was added as competitor. (E) Plot of absorbance (monitored at 320 (▪), 328 nm (□) for bipy-GS_2_ and 340 (•), 335 nm (○) for terpy-GS_2_) vs. the equivalence of metal ion. Line represents best fit for a 1:1 metal:model switch binding ratio.

Similar to the UV/Vis spectra of unsubstituted 2, 2’-bipyridine and 2, 2’:6, 2’-terpyridine,^19^ the absorbance of **bipy-GS_2_** and **terpy-GS_2_** in aqueous solution is sensitive to the pH. The UV/Vis spectra of a 5 μM solution of **bipy-GS_2_** recorded between pH 6 and 10 display two transitions with *λ*_max_ at 295 (ε_295__nm_ 19, 600 m^–1^ cm^–1^) and 245 nm (ε_245__nm_ 15, 200 m^–1^ cm^–1^), assigned as π→π*_1_ and π→π*_2_ transitions.^20^ The analogous **terpy-GS_2_** spectra display a peak with *λ*_max_ 297 nm (ε_297__nm_ 20, 300 m^–1^ cm^–1^) attributed to π→π*_1_, however, the π→π*_2_ transition which occurs around 221 nm, overlaps with that for the peptide bond (Figure 4 and S2). Upon acidification by addition of concentrated HCl, these bands decrease in intensity whilst new bands appear at lower energy. A plot of absorbance as a function of pH allows for an approximation of the associated p*K*_a_ values (see Figure S2).

A similar red-shift of the π→π* bands are observed upon Zn^II^ and Cu^II^ complexation, allowing an apparent binding constant to be determined^10^ taking into account the competitive metal ion binding of the phosphate buffer employed in these experiments.^21^ Aliquots of a stock solution of ZnCl_2_ were titrated into a 5 μM solution of either **bipy-GS_2_** or **terpy-GS_2_** in 20 mM phosphate buffer pH 7.4. This resulted in the steady decrease in the absorbance at 295 (**bipy-GS_2_**) and 297 nm (**terpy-GS_2_**), and an increase in the absorbance at 308 and 320 nm (**bipy-GS_2_**), and 330 and 340 nm (**terpy-GS_2_**), respectively (see Figure 4A and B). A plot of the absorbance as a function of Zn^II^ concentration indicates the formation of a 1:1 complex between Zn^II^ and both the model ligands (see Figure 4E). The observation of an isosbestic point at 303 (**bipy-GS_2_**) and 313 nm (**terpy-GS_2_**) is consistent with the clean formation of the Zn^II^ complex. The extinction coefficient at 320 nm for **bipy-GS_2_** and [Zn(**bipy-GS_2_**)X_n_]^m+^ were determined to be 1, 180 m^–1^ cm^–1^ and 23, 000 m^–1^ cm^–1^, respectively. The extinction coefficient at 340 nm for **terpy-GS_2_** and [Zn(**terpy-GS_2_**)X_n_]^m+^ were estimated to be 800 m^–1^ cm^–1^ and 19, 850 m^–1^ cm^–1^, respectively. Formation constants, log *K*_M_, were calculated to be 6.86 ± 0.04 for [Zn(**bipy-GS_2_**)X_n_]^m+^ and 6.22 ± 0.03 for [Zn(**terpy-GS_2_**)X_n_]^m+^, see Table 1.

**Table 1:** Summary of data obtained for Cu^II^ and Zn^II^ coordination to model switches.

Metal		λ /nm	ε_ML_ /m^–1^ cm^–1^	*K*_app_	*K*_M_	R^2^
Cu^II^	**bipy-GS_2_**	328	1.95 ± 0.01 E +04	6.73 ± 0.27 E +05	3.39 ± 0.14 E +12^a^	0.9987
	**terpy-GS_2_**	335	1.34 ± 0.00 E +04	3.33 ± 0.10 E +06	1.09 ± 0.03 E +08	0.9997
		347	1.53 ± 0.02 E +04	4.21 ± 0.19 E +05	1.38 ± 0.06 E +07	0.9988
Zn^II^	**bipy-GS_2_**	320	2.27 ± 0.02 E +04	1.21 ± 0.10 E +06	7.30 ± 0.61 E +06	0.9965
	**terpy-GS_2_**	340	1.99 ± 0.03 E +04	2.77 ± 0.15 E +05	1.67 ± 0.09 E +06	0.9983

Titration performed with glycine (20 mM) as competitor in addition to phosphate buffer.

The analogous titration performed with CuCl_2_, resulted in a decrease in the absorbance at 295 nm (**bipy-GS_2_**) and 297 nm (**terpy-GS_2_**), accompanied by an increase in the absorbance at 317 and 328 nm (**bipy-GS_2_**) and at 335 and 347 nm (**terpy-GS_2_**), assigned to the formation of [Cu(**bipy-GS_2_**)X_n_]^m+^ and [Cu(**terpy-GS_2_**)X_n_]^m+^, respectively. Monitoring the absorbance at 328 nm (**bipy-GS_2_**) and 335 nm (**terpy-GS_2_**) and plotting this as a function of CuCl_2_ equivalence, is also consistent with the formation of a 1:1 complex with extinction coefficients estimated to be ε_317_ 19, 500 m^–1^ cm^–1^ ([Cu(**bipy-GS_2_**)X_n_]^m+^) and ε_335_ 13, 400 m^–1^ cm^–1^, ε_347_ 15, 300 m^–1^ cm^–1^ ([Cu(**terpy-GS_2_**)X_n_]^m+^). The isosbestic points at 305 (**bipy-GS_2_**) and 315 nm (**terpy-GS_2_**) are again consistent with a single equilibrium (see Figure 4C and 4D), Formation constants, log *K*_M_, were estimated to be 12.5 ± 0.1 for [Cu(**bipy-GS_2_**)X_n_]^m+^ and two slightly different values of 8.04 ± 0.01 (A_335_) and 7.14 ± 0.02 (A_347_) were obtained for [Cu(**terpy-GS_2_**)X_n_]^m+^, depending on the wavelength monitored, see Table 1.

**Figure 5:**
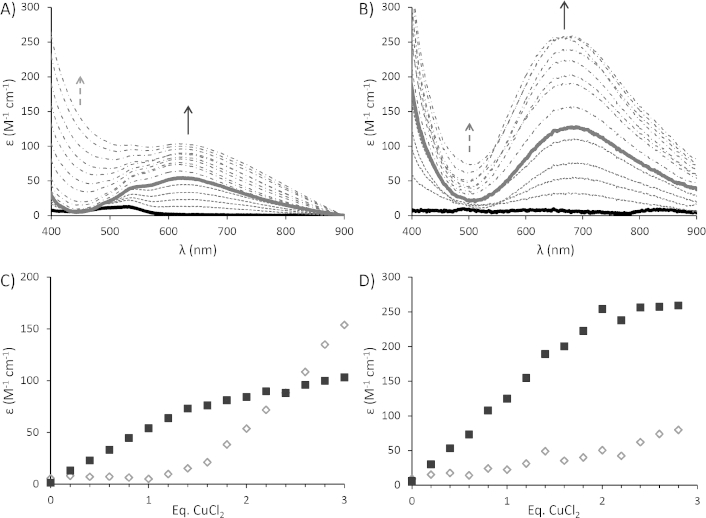
UV spectra for the Cu^II^ titration of model switches. CuCl_2_ was added to solutions containing either (A) 350 μM bipy-GS_2_ or (B) 100 μM terpy-GS_2_ buffered at pH 7.4. Plot of absorbance monitored at (C) 622 (▪) and 440 nm (◊) for bipy-GS_2_ and (D) 670 (▪) and 500 nm (◊) for terpy-GS_2_, vs. the equivalence of Cu^II^.

The addition of CuCl_2_ to solutions of **bipy-GS_2_** and **terpy-GS_2_** buffered at pH 7.4 was also accompanied by transitions between 400–900 nm. Aliquots of a stock solution of CuCl_2_ titrated into a more concentrated 350 μM solution of **bipy-GS_2_**, resulted in an increase in the absorbance at 622 nm up to 1.5 equivalents of Cu^II^. Further addition of CuCl_2_ led to only a small increase in the absorbance at 622 nm and an increase at 440 nm, consistent with addition of CuCl_2_ to the blank buffered solution (see Figure 5A). An analogous titration of CuCl_2_ into a 100 μM solution of **terpy-GS_2_** buffered at pH 7.4, resulted in an increase in the absorbance at 675 nm up to 2.0 equivalents of Cu^II^, see Figure 5B, which blue-shifted shifted slightly to 670 nm on addition of between 1.0 and 1.5 equivalents of Cu^II^. No further changes were observed upon addition of between 2.0 and 3.0 equivalents of CuCl_2_.

### ^1^H NMR Spectroscopy

**Figure 6:**
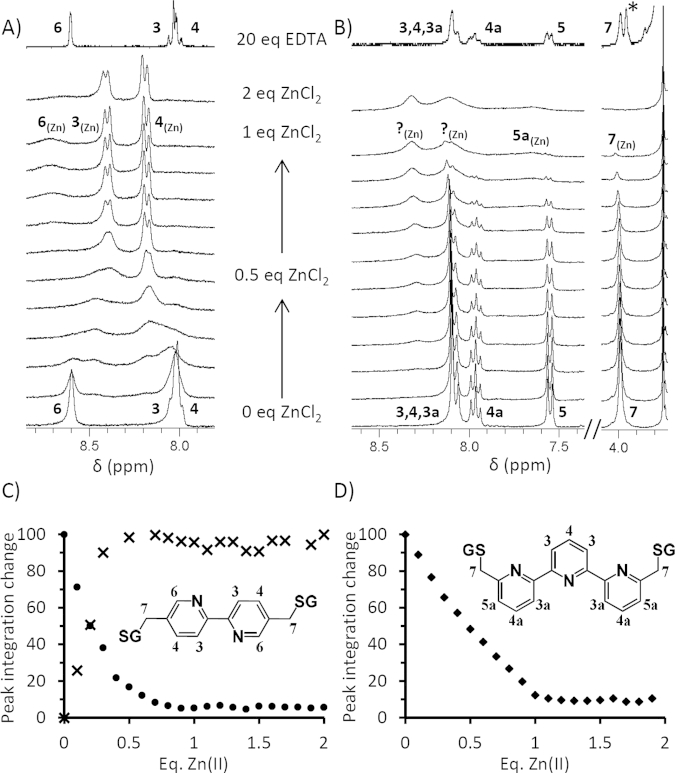
^1^H NMR Zn^II^ titration of bipy-GS_2_ and terpy-GS_2_ in solution buffered at pD 7.4 for (A) bipy-GS_2_ and (B) terpy-GS_2_. Plot of change in percentage peak integration as a function of equivalence of ZnCl_2_ for (C) 8.04–8.00 ppm resonances for H^3^ and H^4^ of the free model switch, bipy-GS_2_ (•), as well as for the new peak at *δ* = 8.20 ppm for H^4^ of the Zn-bipy-GS_2_ adduct (X), and (D) the terpy-GS_2_ methyl singlet (♦) at *δ* = 3.99 ppm. The peak at *δ* = 3.96 ppm labelled with *, was attributed to the ^13^C satellite relative to the -N-CH_2_-COOH signal of EDTA centred at *δ* = 3.72 ppm.

The ^1^H NMR spectrum of a 5 mM solution of **bipy-GS_2_** in D_2_O at pD 1, displays two singlets at *δ* = 8.83 and 8.42 ppm in the aromatic region, integrating to two and four protons, respectively. In contrast, one singlet at *δ* = 8.59 ppm and two overlapping doublets at 8.04 and 8.00 (AB pattern), each integrating to 2 protons, are observed on raising the pD to 7.4 (see Figure S3). A titration of ZnCl_2_ into a 5 mM solution of **bipy-GS_2_** in D_2_O buffered at pD 7.4, results in the broadening and decrease in intensity of the peaks at *δ* = 8.59, 8.04 and 8.00 ppm, and the appearance of new peaks at 8.66 (broadened, H^6^), 8.42 (doublet, H^3^) and 8.20 (doublet, H^4^) ppm on addition of 1.0 equivalent of ZnCl_2_ (see Figure 6A). A plot of peak integration of the overlapping doublets (8.04 and 8.00 ppm) as a function of equivalence of ZnCl_2_ indicates a 1:2 Zn:**bipy-GS_2_** ratio. Similarly a plot of peak integration for the resulting doublet for the Zn-**bipy-GS_2_** adduct at *δ* = 8.20 ppm, also plateaus at 0.5 equivalence ZnCl_2_ consistent with a 1:2 Zn:**bipy-GS_2_** ratio (see Figure 6C). After 0.5 equivalents of ZnCl_2_ have been added the peaks at *δ* = 8.59, 8.04 and 8.00 ppm appear to have been replaced with broad new peaks at *δ* = 8.42 and 8.20 ppm. Upon addition of between 0.5 and 1.0 equiv. ZnCl_2_ these peaks sharpen into doublets and a broad peak attributed to H^6^ appears at higher frequency (δ = 8.66 ppm). Only very small changes are observed on addition of between 1.0 and 2.0 equivalents of ZnCl_2_ (see Figure 6A).

At acidic pD (ca. 1) the ^1^H NMR spectrum of **terpy-GS_2_** recorded in D_2_O displays 5 aromatic signals (all doublets of doublets) at 8.68 (2H^3a, b^), 8.67 (2H^4a, b^), 8.59 (2H^3^), 8.47 (1H^4^) and 8.17 ppm (2H^5a, b^), which were assigned using COSY and NOESY NMR (see Figures S4 and S5). The COSY spectrum displays cross-peaks between H^3^ <–> H^4^ and H^4a^ <–> H^5a^ (n.b. H^3a^ and H^4a^ are too close, so the cross-coupling overlaps with the diagonal peaks), see Figure S5A. In contrast, NOESY NMR recorded under similar conditions, displays an additional H^3a^ <–> H^3^ inter-ring coupling (see Figure S5B).

On raising the pD to 7.4, three aromatic resonances are observed at 8.08 (1 H^4^, 2H^3^, 2H^3a^), 7.96 (2H^4a^) and 7.55 ppm (2H^5a^). A titration of ZnCl_2_ into a 5 mM solution of **terpy-GS_2_** in D_2_O buffered at pD 7.4, resulted in the decrease in intensity of the peaks at *δ* = 8.08, 7.96 and 7.55 ppm, and the appearance of new broad peaks at *δ* = 8.31, 8.13 and 7.65 ppm. This is accompanied by a decrease in the intensity of the singlet at *δ* = 3.99 ppm assigned to the CH_2_-pyridinyl group. A plot of the peak integration for the singlet at *δ* = 3.99 ppm, as a function of ZnCl_2_ concentration (Figure 6B and D), is consistent with formation of a 1:1 complex between Zn^II^ and **terpy-GS_2_**.

In both cases, addition of excess EDTA (20 equiv. with respect to ZnCl_2_) resulted in ^1^H NMR spectra which are in good agreement with those of **bipy-GS_2_** and **terpy-GS_2_** recorded in the absence of ZnCl_2_ (see Figure 6A and 6B).

### Ion Mobility Spectrometry (IMS) Mass Spectrometry (MS)

**Figure 7:**
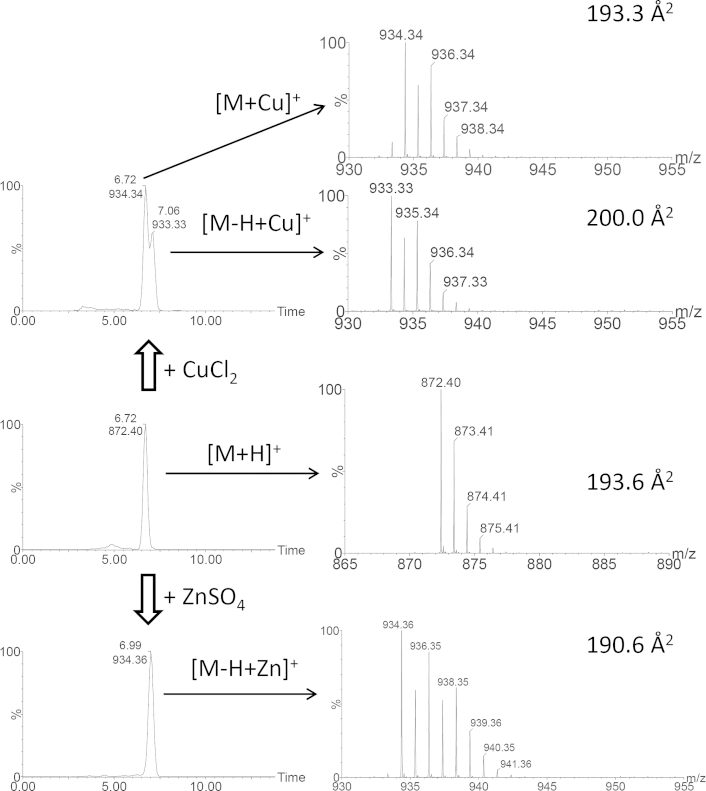
Ion-mobility mass spectrometry spectra on addition of Cu^II^ or Zn^II^ to terpy-GS_2_. Drift times (ms) for [M + H]^+^ ion of terpy-GS_2_; [M+Cu]^+^ and [M – H+Cu]^+^ ions of terpy-GS_2_ in the presence of 1 equivalence CuCl_2_; and the [M – H+Zn]^+^ ion of terpy-GS_2_ in the presence of 1 equivalence ZnSO_4_. Associated ESI-MS spectra of these ions, and their calculated cross sections.

Ion mobility spectrometry (IMS) coupled to electrospray ionisation (ESI) mass spectrometry (MS), has been used to examine the model switches, **bipy**-**GS_2_** and **terpy**-**GS_2_**, in the absence and presence of CuCl_2_ and ZnSO_4_. A single species is detected for the **terpy**-**GS_2_** model switch with a drift time (DT) of 6.72 ms, which is consistent with [M + H]^+^ and for which a collision cross-section (CCS) of 193.6 Å^2^ has been calculated (see Figure 7). When **terpy**-**GS_2_** is combined with 1.0 equivalent of CuCl_2_ it forms a split peak, indicating the presence of 2 species, in a 60:40 ratio, separated by 1 Da. Extracting these and looking more closely at their isotopic distributions, it appears that these are consistent with [M+Cu]^+^ and [M – H+Cu]^+^ (see Figure 7). The [M+Cu]^+^ species displays a near identical DT and CCS when compared to **terpy**-**GS_2_** in the absence of CuCl_2_. However, the [M – H+Cu]^+^ species with a DT of 7.06 ms, shows a small change in the calculated CCS (200.0 Å^2^).

A single species is also detected for the **bipy**-**GS_2_** model switch in the absence of metal ions, with a DT of 6.02 ms, consistent with [M + H]^+^ and a CCS of 179.3 Å^2^ (see Figure S6). Similar results are obtained when **bipy**-**GS_2_** is combined with 1.0 equivalent of CuCl_2_, i.e. 2 species separated by 1 Da and consistent with [M+Cu]^+^ and [M – H+Cu]^+^. The first species, [M+Cu]^+^, displays a similar DT and CCS to **bipy**-**GS_2_**, and the second species, [M – H+Cu]^+^, displays a longer DT of 6.58 ms and a slightly larger calculated CCS (190.4 Å^2^). However, the intensities of these two species is extremely different than for **terpy**-**GS_2_**, almost exclusively forming the [**bipy**-**GS_2_-**H+Cu]^+^ species (5:95) (see Figure S6). Analogous experiments conducted with higher ratios of CuCl_2_ (1.4 and 1.9 equivalence) yielded almost identical results.

Similar experiments performed with ZnSO_4_, show the formation of a species consistent with [M – H+Zn]^+^ with a slightly longer drift time (6.99 ms [**terpy**-**GS_2_**-H+Zn]^+^; 6.30 ms [**bipy**-**GS_2_**-H+Zn]^+^) than the model switch in the absence of ZnSO_4_, and a very similar CCS (190.6 Å^2^ [**terpy**-**GS_2_**-H+Zn]^+^; 178.1 Å^2^ [**bipy**-**GS_2_**-H+Zn]^+^). In the case of **terpy**-**GS_2_** this is the only species detected, however, two additional species are detected for **bipy**-**GS_2_** in the presence of ZnSO_4_ (see Figure S6). One is consistent with a *m*/*z* of 851 and a DT of 6.02 ms. The second with *m*/*z* 855 (DT 4.09 ms), and with an isotopic distribution consistent with a +2 charge ion. However, these species were not subjected to further characterisation.

## Discussion

### Zn^II^ Coordination of Model Switches

Titration of Zn^II^ into a 5 mM solution of **terpy-GS_2_** monitored by ^1^H NMR, is consistent with a 1:1 binding ratio (see Figure 6B). Similar CD and UV/Vis titrations performed at micromolar concentrations, were also consistent with the formation of a 1:1 [Zn(**terpy-GS_2_**)X_n_]^m+^ complex (where X can be an exogenous ligand such as a water molecule, hydroxide or chloride).

In contrast, the titration of Zn^II^ into a 5 mM solution of **bipy-GS_2_** indicates the formation of a 2**bipy-GS_2_**:1Zn^II^ complex, possibly followed by formation of a 1**bipy-GS_2_**:1Zn^II^ complex. This is in stark contrast to the analogous titrations (CD, UV) recorded under more dilute (14 times and 1000 times respectively) and biologically relevant conditions, suggesting that the formation of a 2:1 complex with **bipy-GS_2_** only occurs at high concentrations, and that the 1:1 [Zn(**bipy-GS_2_**)X_n_]^m+^ species dominates under more dilute conditions.

The relatively featureless CD spectra of the model switches, are altered dramatically upon coordination of Zn^II^. Titrations of **bipy-GS_2_** and **terpy-GS_2_**, display chiral induced CD signals relative to the bipy and terpy π→π* bands (250–400 nm range). The cotton effect induced upon Zn^II^ addition is opposite for **terpy-GS_2_** and **bipy-GS_2_** (see Figure 2). This indicates that the lower energy transition for [Zn(**terpy-GS_2_**)X_n_]^m+^ might arise from π→π* electronic absorptions involving molecular orbitals composed mainly from atomic orbitals from atoms composing the central pyridine ring, as previously suggested.^19^ In contrast, the second lower energy transition (centred at 287 nm) for [Zn(**terpy-GS_2_**)X_n_]^m+^ could involve orbitals comprising contributions mainly from the external pyridines, and is therefore more similar to the lower energetic absorption band for [Zn(**bipy-GS_2_**)X_n_]^m+^ (see Figure 2). These observations are consistent with formation of a 1:1 complex with the Zn^II^ coordinated to the intended polypyridine chelate of the model switches. Glutathione units might also contribute to the coordination sphere, but this currently remains unclear.^22^

### Cu^II^ Coordination of Model Switches

The shift in the **bipy-GS_2_** and **terpy-GS_2_** π → π* band (200–400 nm range) in the UV/Vis spectra upon addition of either Cu^II^ or Zn^II^, is consistent with a ca. 1:1 ratio in all cases. However, CD spectra suggest that the complexation of Cu^II^ is more complicated, and involves the formation of two different Cu^II^ complexes with differing contributions to the metal-bound π → π* bands. This is not obvious in the UV/Vis spectra in this range, but is observed for Cu^II^ complexation to both **bipy-GS_2_** and **terpy-GS_2_** by CD, due to exciton effects (see Figure 3).^23^–^25^ The CD titration of Cu^II^ into **bipy-GS_2_**, results in chiral induced signals relative to the bipy π→π* band (250–400 nm range) up to one equivalent consistent with the formation of a 1:1 complex involving coordination through the pyridine units. However, the CD titration indicates that this is followed by the formation of a 1.5Cu:1**bipy-GS_2_** complex, as a result of a reduction in these induced CD signals. In contrast, the analogous CD titration with **terpy-GS_2_** did not result in the formation of induced CD signals relative to the terpy π→π* band up to one equivalent of Cu^II^. However, it is consistent with the initial formation of a 1:1 complex, followed by a 2Cu:1**terpy-GS_2_** complex (see Figure 3B).

Similarly, monitoring of the metal-to-ligand charge-transfer (MLCT) band by UV/Vis spectroscopy (400–900 nm range), which appears upon coordination of Cu^II^, indicates the final formation of a 1.5Cu:1 **bipy-GS_2_** and 2Cu:1**terpy-GS_2_** complex (see Figure 5C and 5D). The MLCT for Cu^II^ coordination to the model switches in a 1Cu:1**bipy-GS_2_** and 1Cu:1**terpy-GS_2_** ratio, (**bipy-GS_2_**: *λ*_max_ = 620 nm, ε_620_ = 51 m^–1^ cm^–1^; **terpy-GS_2_**: *λ*_max_ = 675 nm, ε_675_ = 131 m^–1^ cm^–1^) is consistent with previous reports for the formation of [Cu(**bipy-GS_2_**)(OH)_2_] and [Cu(**terpy-GS_2_**)(H_2_O)*_n_*]^2+^, as only a small difference in *λ*_max_ is expected for substitution with chloride.^14^,^26^ Small MLCT shifts are observed upon addition of between 1–1.5 equiv. Cu^II^ (**bipy-GS_2_**) or 1–2 equiv. Cu^II^ (**terpy-GS_2_**), consistent with only minor changes to the Cu ion coordination environment. Similarly the lack of contribution from the π→π* on formation of the 1.5Cu:1 **bipy-GS_2_** and 2Cu:1**terpy-GS_2_** complexes, is consistent with no significant change to the polypyridine coordination chemistry with respect to the analogous 1:1 complexes.

We hypothesise that on formation of the initial 1:1 complexes, the Cu^II^ coordinates to the bipyridine or terpyridine ligand, and either exogenous water (or hydroxide) molecules or groups from the glutathione units (for example the N- or C-termini, amino acid side chains or amide bonds^27^). However, the formation of the 1.5Cu:1 **bipy-GS_2_** complex could involve the formation of a new intermolecular Cu^II^ coordination site between two Cu(**bipy-GS_2_**) complexes. Whereas on formation of the 2Cu:1**terpy-GS_2_** complex, the second Cu^II^ could be coordinated exclusively by the glutathione units.

### Binding Constants

The shift of the π→π* band was used to estimate the Cu^II^ and Zn^II^ binding constants to the model switches. Titrations were performed at low micromolar concentrations, and where necessary a competitor was introduced. Plots of the lower energy absorbance maxima for the resulting complexes π→π* transition^28^ vs. metal ion concentration, were fit to a 1:1 model, as this shift corresponds to formation of the 1:1 polypyridine:metal complex (see Figure 4E). The binding constants reported in Table 1 are in good agreement with those reported previously for related ligands using similar methods.[10, 11, 29–32] Our model compounds, **bipy-GS_2_** and **terpy-GS_2_**, display higher affinity for Cu^II^ than for Zn^II^, consistent with previous reports;^29^ and **bipy-GS_2_** displays a higher affinity for both Cu^II^ and Zn^II^ than **terpy-GS_2_**. The latter observation is consistent with lowering of the binding constant for **terpy-GS_2_** resulting from the strain introduced by substitutions at position 6- and 6”-, as previously reported for polypyridine amino-acid conjugates.^10^ In the case of **terpy-GS_2_**, fitting the data for the two π→π* transitions as a function of Cu^II^ concentration lead to different affinities, related by a factor of 10 (see Table 1). We postulate that these are due to the different Cu^II^ coordination environments, where the two species contribute differently to the absorbance at 335 and 347 nm (fitted to obtain the formation constants).

### Conformational Study of the Model Switches

#### UV/Vis Spectroscopy

*Nakamato* first studied the pH dependence of 2, 2-bipyridine and 2, 2’:6’,2”-terpyridine in water, and demonstrated that at low pH, free bipy and terpy display similar absorption profiles to those of the metal complexes, consistent with a *cisoïd*- conformation. However, the π→π* bands shift to higher energy on increasing the pH, and resembles those recorded in organic solvents, consistent with the *transoïd*-conformation.^19^,^29^ It was hypothesised that deprotonation of the pyridinyl ring on increasing the pH, resulted in a conformational transition of the bipy and terpy from *cisoïd*- to *transoïd*-, and has been supported more recently by theoretical studies.^33^–^35^ The equilibria are characterised by clear isosbestic points, and p*K*_a_ values of 4.44 (bipy), 2.59 and 4.16 (terpy) were reported.^20^ Similar pH titrations performed on our model compounds, **bipy-GS_2_** and **terpy-GS_2_**, are consistent with the reported protonation constants (Figure S2) and the presence of a predominantly *transoïd*- conformation at physiological pH. These results importantly illustrate that introduction of short peptides into the design, does not alter the pH dependent behaviour of these polypyridine switching units as monitored in solution under dilute micromolar conditions by UV/Vis spectroscopy. Therefore, unlike *Kelly* and co-workers^12^ our model switches adopt the *transoïd* conformation at neutral and physiologically relevant pH. This could be due to coupling through an amino acid side chain, rather than introduction into the peptide backbone. Notably the addition of Cu^II^ and Zn^II^ to our **bipy-GS_2_** and **terpy-GS_2_** model compounds at pH 7.4, lead to a shift of π→π* bands toward higher energy, consistent with a *transoïd*- to *cisoïd*- conformational transition. Importantly these results indicate that metal ions can be used to control our model switches under biologically relevant conditions (under dilute conditions in aqueous solution and at a physiologically relevant pH).

#### ^1^H NMR Spectroscopy

The ^1^H NMR spectrum of **bipy-GS_2_** recorded under acidic conditions is very different from that recorded at neutral pD. A single resonance, attributed to H^3^ and H^4^ of **bipy-GS_2_**, is observed at pD 1, however, an AB pattern where the two overlapping doublets are located at a lower chemical shift, is observed at pD 7.4. Addition of ZnCl_2_ to a 5 mM solution of **bipy-GS_2_** at pD 7.4 results in new broad peaks which indicate a species in slow/intermediary exchange on the NMR time-scale. A plot of peak integration as a function of Zn^II^ equivalence is consistent with the formation of a 2**bipy-GS_2_**:1Zn complex. This spectrum at 0.5 equivalents Zn^II^ does not display any signal assigned to H^6^, most likely due to signal broadening as a result of the clash between the two bipyridine in the binary complex. However a broad resonance assigned to H^6^ reappears on addition of more ZnCl_2_ (between 0.5 and 1 equivalent) and sharpens in the presence of excess Zn^II^ (5 and 10 equivalents), which may be consistent with conversion of the 2**bipy-GS_2_**:1Zn complex into a 1**bipy-GS_2_**:1Zn complex.^36^,^37^

Theoretical studies suggest that even though monoprotonated bipyridine and bidentate metal complexes of bipyridine have energy minima with similar conformations (*cisoïd*), flexibility around the axial bond of bipyridine is much higher in the monoprotonated bipyridine compared to the metal complexes.^38^ In fact, the difference in potential energy separating *cisoïd* and *transoïd* conformations has been reported to be comparable for the monoprotonated bipyridine and the free bipyridine.^33^,^34^,^38^ This could account for the similarity of resonances assigned as H^3^ and H^4^ in spectra of **bipy-GS_2_** recorded at acidic and neutral pD, which in turn differ from those recorded for the Zn^II^ complex (see Figure S3). NMR spectra of bipyridine or derivatives where H^3^ and H^4^ resonances overlap have previously been recorded in aqueous solution at both acidic^36^,^39^ and physiological^36^,^40^ pH, however, this is not exclusively the case.^39^ Interpretation of bipyridine conformation based on NMR chemical shift can therefore lead to contradictory results. For example, theoretical studies suggest that H^3^ are deshielded in the *transoïd* (cation free) bipyridine due to the close proximity with the nitrogen on the second ring.^13^ Cation binding to bipyridine is expected to result in deshielding of aromatic resonances. In contrast, H^3^ can display only a moderate deshielding,^39^ or shielding^41^ upon *transoïd* to *cisoïd* conformational transition, as deshielding effects on H^3^ from the proximal nitrogen are lost.^36^,^42^ Therefore an attractive method by which to assign the **bipy-GS_2_** conformation is by monitoring intra-ring coupling by NOESY NMR,^43^ however, this is only possible for an asymmetric bipyridine for which H^3^ peaks are inequivalent, and so cannot be applied to **bipy-GS_2_**.

In contrast, NOESY can be applied to **terpy-GS_2_**. At pD 1, the cross-peak observed between H^3^ and H^3a^ in the NOESY spectrum is consistent with at least half of the terpyridine adopting a *cisoïd* conformation (see Figure S5B). The ^1^H NMR spectrum of **terpy-GS_2_** recorded at pD 7.4 is different from that recorded at acidic pD, and is not suitable for determination of intra-ring coupling as the resonances for H^3^ and H^3a^ overlap. Upon raising the pD from 1 to 7.4, all aromatic signals move to lower frequency, and proton signals relative to external pyridine (H^3a^, H^4a^ and H^5a^) experience higher shielding than their counterpart (H^3^ and H^4^), consistent with a transition from mixed (*cis-trans*) conformation to a *transoïd* (*trans-trans*) conformation.^44^ Addition of ZnCl_2_ lead to formation of broad resonances for a 1**terpy-GS_2_**:1Zn complex in slow/intermediary exchange on the NMR timescale. Signals are generally broad and difficult to assign, but integration of the three signals indicates that the spectrum is different from that recorded for **terpy-GS_2_** both at acidic and neutral pD, and could be consistent with the *cisoïd* conformation of a terpyridine metal complex.^29^,^31^

#### Ion Mobility Spectrometry (IMS) Mass Spectrometry (MS)

The transition between *cisoïd*- and *transoïd*- polypyridine conformers is not the only conformational transition involved in our peptide conjugates of bipy or terpy. The thioether linkage and the peptide backbone will contribute to the overall global structure of the molecule and is likely to dominate when considering the molecules collisional cross-section (CCS). In an attempt to monitor the overall conformation of the model switches in the gas-phase, we have employed ion mobility spectrometry (IMS) couple to ESI-MS to study **bipy-GS_2_** and **terpy-GS**_**2**,_ in the absence and presence of Cu^II^ and Zn^II^. In the absence of any metal ions these measurements found that both **bipy-GS_2_** and **terpy-GS_2_** were detected in the protonated form in the gas-phase (samples prepared at pH 6.7), which is associated with the *cisoïd* (bpy) and a mixed *cis*-*trans* conformation (terpy) in solution. Therefore, it is possible that any changes observed in the CCS can be attributed to changes in the orientation of the glutathione units, rather than a *transoïd- cisoïd* conformational transition.

As expected **bipy-GS_2_** traverses the mobility T-Wave with a short drift time (DT) and is consistent with a smaller CCS when compared with the **terpy-GS_2_** model. Coordination of Zn^II^ to **bipy-GS_2_** or **terpy-GS_2_** leads to the formation of a species consistent with [M – H+Zn]^+^ with a longer drift time but similar CCS to the model switch in the absence of Zn^II^. The ion-mobility spectrum recorded for **bipy-GS_2_** in the presence of Zn^II^ indicates the formation of at least three different species. We have not been able to assign the remaining two, but they may involve partial decomposition of the complex and cluster formation in the gas-phase.

The addition of Cu^II^ resulted in the detection of two different species by IMS. One of these Cu adducts could be consistent with Cu^I^ replacing the proton and has a near identical drift time and CCS to the model switch in the absence of metal ions. The second Cu adduct has been assigned as [M – H+Cu]^+^ (consistent with Cu in the +2 oxidation state), is detected after a longer drift time and has a slightly larger CCS than the model switch in the absence of metal ions. Comparison of the CCS measurements suggests that there is potentially some structural difference, which we propose is due to complexation of the bipyridine/terpyridine and subsequent repositioning and potential coordination of the glutathione units. Formation of Cu^II^ complexes in the gas-phase involving a mixture of bipyridine and peptide ligands, have previously been described.^45^ Both the Cu adducts are formed in nearly equal amounts on coordinating to the **terpy-GS_2_** model switch (ca. 60:40), however, the second Cu adduct, [M – H+Cu]^+^, is almost exclusively formed on coordination to **bipy-GS_2_** (ca. 5:95). This would be consistent with formation of polypyridine-peptide complexes with different stability constants in the gas-phase,^45^ similar to the formation of a more stable **bipy-GS_2_** complex with Cu^II^ in aqueous solution. The different CCS for the various species formed (Cu/Zn) could be due to the different preferred metal ion coordination geometries. Similar metal ion conformational dependence in the gas-phase was described previously in an IMS study of the Gramicidin peptide.^46^

## Conclusions

In conclusion, two model switches for the metal dependent spatial alignment of protein fragments in synthetic biology, have been prepared and studied in aqueous solution by UV/Vis, CD, NMR and IMS-MS. The model switches, **bipy-GS_2_** and **terpy-GS_2_**, contain a polypyridine linker (either based on 5, 5’-disubstituted-2, 2’-bipyridine or 6, 6”-disubstituted-2, 2’:6’,2”-terpyridine) coupled through the native Cys side chain of the tripeptide glutathione. This approach can therefore be extended to the coupling of larger natural protein fragments. Introduction of the peptide substituents was shown to have little effect on the polypyridine pH dependence, adopting the predominantly *transoid* conformation in aqueous solution at neutral pH.

Variations to the polypyridine design resulted in different Cu^II^ and Zn^II^ coordination properties. Zn^II^ coordination involves formation of a 1:1 complex with both **bipy-GS_2_** and **terpy-GS_2_** under biologically relevant conditions (micromolar concentrations and neutral pH). The coordination of Cu^II^ was more complicated and appears to initially involve the formation of a 1:1 complex followed by a 1.5Cu:1**bipy-GS_2_** and 2Cu:1**terpy-GS_2_** complex. Cu^II^ binding constants were found to be higher than those obtained for Zn^II^, and the sterically less strained **bipy-GS_2_** forms more stable complexes with both metal ions than **terpy-GS_2_**. Metal ion complexation was further investigated by IMS-MS, and changes to CCS upon metal ion coordination were found to be similar for both model switches.

Though polypyridine metal ion complexation has been extensively studied in the literature in organic solvents, this has not received the same kind of attention in water. This work therefore represents an important contribution towards their use in biologically relevant systems. Work is now on-going to investigate the use of these conformational switches with larger peptide fragments attached to the polypyridyl ligand, to evaluate if the metal ion dependent spatial rearrangement can be exploited to align these fragments in order to achieve enhanced biomolecular recognition.

## Experimental Section

NaHCO_3_, Na_2_SO_4_, NaNO_2_, NaOH, KOH, mono- and dihydrogen potassium salts, Tris Base, ethylene diamine tetra acetic acid (EDTA), chloroform (CHCl_3_), methanol (CH_3_OH), ethanol, tetrahydrofuran (THF), toluene, dichloromethane (CH_2_Cl_2_), acetonitrile, dioxane, water (HPLC grade), hydrobromic acid 48 %, HCl 32 %, ammonia 35 %, bromine, were all obtained from Fisher Scientific. 5, 5’-Dimethyl-2, 2’-bipyridine, N-butyllithium 1.6 m in hexane, ethyl ether, copper chloride (CuCl_2_), L-glycine, were obtained from Sigma-Aldrich. 2-Bromopicoline, 2, 6-dibromopyridine, tetrakis-(triphenylphosphine) palladium, trifluoroacetic acid (TFA), tributyltin chloride, zinc chloride (ZnCl_2_) were obtained from BOC. N-Bromosuccinimide was obtained from Alfa-Aesar, and L-glutathione from Fluka (> 97 % pure by HPLC, as a sum of enantiomers). Deuterated solvents (CDCl_3_ and D_2_O) were obtained from Cambridge Isotope Laboratory Inc.

HRES and ES-TOF MS were recorded with a Micromass LCT TOF spectrometer equipped with a 3000 V capillary voltage, and a cone voltage of 35 V. GC–MS were recorded with a Waters GCT Premier Micromass equipped with an Electronic Impact (EI) probe.

### Synthesis of 5, 5′-Dibromomethyl-2, 2′-bipyridine

5, 5’-Dimethyl-2, 2’-bipyridine (0.389 g, 2.11 mmol), N-bromosuccinimide (0.756 g, 4.23 mmol) and azobisisobutyronitrile (10 mg, 0.06 mmol) were dissolved in dichloromethane (20 mL), refluxed using a 500 W halogen lamp, and the reaction progress monitored by TLC (SiO_2_, eluent: CH_2_Cl_2_/CH_3_OH (9/1)). After 4 hours, more N-bromosuccinimide (0.375 g, 2.10 mmol) was added and the reaction was refluxed for a further 2 hours. The mixture was allowed to cool to room temperature and the solution extracted with 0.1 m aqueous NaHCO_3_ (5 × 20 mL). The organic layer was dried with Na_2_SO_4_, filtered, and concentrated in vacuo. The solid was re-dissolved in 15 mL of a 50/50 mix CHCl_3_/CH_3_OH, and the resulting solution stored in the freezer overnight. A white solid precipitate was collected by filtration, air-dried, and recrystallized from CHCl_3_, to afford white crystals (0.219 g, 30 %). **^1^H NMR** (300 MHz, CDCl_3_): 8.69 (d, ^3^*J*_H6–H4_ = 2.1 Hz, 2 H, H^6^), 8.41 (d, ^3^*J*_H3–H4_ = 8.2 Hz, 2 H, H^3^), 7.86 (dd, ^3^*J*_H4–H6_ = 2.3, ^3^*J*_H4–H3_ = 8.2 Hz, 2 H, H^4^), 4.54 (s, 4 H, H^7^). **^13^C NMR ^1^H decoupled** (100 MHz, CDCl_3_): 155.4 (C^2^), 149.4 (C^6^), 137.9 (C^4^), 134.1 (C^5^), 121.4 (C^3^), 29.6 (C^7^). **HRES-TOF** (CH_2_Cl_2_): calculated mass for C_12_H_10_N_2_Br_2_Na = 362.9108; measured = 362.9126 ([M + Na]^+^, 100 %).

### Synthesis of 2-Bromo-6-methylpyridine

2-Bromopicoline (10.8 g, 115 mmol) in hydrobromic acid (40 mL, 48 %) was cooled to –20 °C in an ethanol bath. Bromine (14.4 mL, 280 mmol) was added dropwise, and the suspension was stirred for 90 min at –20 °C. An aqueous solution of NaNO_2_ (30 mL, 8.9 m, 268 mmol) was added dropwise, and the solution was allowed to warm to room temperature over 2 hours with stirring. The mixture was re-cooled to –20 °C, and a cool NaOH aqueous solution (110 mL, 16.5 M, 1.81 mol) added slowly, while maintaining the temperature below –10 °C. The mixture was allowed to warm to room temperature over the course of an more hour with continuous stirring. The mixture was then extracted with ethyl acetate and the organic layer dried with Na_2_SO_4_, filtered, and concentrated in vacuo. The dark oil was then purified by Kugelrohr distillation to yield a colourless oil (9.906 g, 50 %). **^1^H NMR** (300 MHz, CDCl_3_): 7.43 (t, ^3^*J*_H4–H5_ = ^3^*J*_H4–H3_ = 7.7 Hz, 1 H, H^4^), 7.29 (d, ^3^*J*_H5–H4_ = 7.9 Hz, 1 H, H^5^), 7.10 (d, ^3^*J*_H3–H4_ = 7.4, 1 H, H^3^), 2.54 (s, 3 H, H^7^). **^13^C NMR ^1^H decoupled** (100 MHz, CDCl_3_): 160.2 (C^6^), 141.5 (C^2^), 138.7 (C^4^), 125.2 (C^5^), 122.2 (C^3^), 24.3 (C^7^). **HRES-TOF** (CH_2_Cl_2_): calculated exact mass for C_6_H_6_^79^BrN = 170.9684; measured = 170.9686: **ES-TOF** *m*/*z* = 171.0 ([M^.+^], 50 %), 92.0 ([M-Br]^+^, 100 %), 65.0 ([M-C_2_H_3_Br]^+^, 75 %).

### Synthesis of 2-Tributylstannyl-6-methylpyridine

A solution of 2-bromo-6-methylpyridine (4.63 g, 27 mmol) in dry THF (20 mL) was cooled to –60 °C, 27 mL of n-butyllithium in hexane (1.1 m, 30.1 mmol) added dropwise, and the solution was stirred for 2 hours at 0 °C. Tributyltin chloride (8.9 mL, 32.8 mmol) was slowly added and the solution allowed to return to room temperature over 20 minutes with continuous stirring. Water (30 mL) was added into the reaction mixture, and phases were separated. The organic phase was washed with water (3 × 30 mL), and the combined aqueous layers washed with ethyl ether (4 × 30 mL). The combined organic phases were dried with Na_2_SO_4_, filtered, and concentrated in vacuo to afford a black oil (9.534 g, 92 %). The crude product was determined to be ca. 95 % pure by GC analysis and so used directly in the following synthesis. **^1^H NMR** (300 MHz, CDCl_3_): 7.36 (t, ^3^*J*_H4–H5_ = ^3^*J*_H4–H3_ = 7.5 Hz, 1 H^4^), 7.17 (d, ^3^*J*_H4–H5_ = 7.3 Hz, 1 H, H^5^), 6.96 (d, ^3^*J*_H4–H3_ = 7.9, 1 H, H^3^), 2.54 (s, 3 H^7^), 1.56 (m, 6 H, H^9^), 1.33 (m, 6 H, H^10^), 1.09 (m, 6 H, H^8^), 0.88 (t, ^3^*J*_H11–H10_ = 7.2, 9 H, H^11^). **^13^C NMR ^1^H decoupled** (100 MHz, CDCl_3_): 173.2 (C^6^), 158.7 (C^2^), 133.3 (C^4^), 129.5 (C^5^), 121.6 (C^3^), 29.2 (C^9^), 27.5 (C^10^), 25.1 (C^7^), 13.8 (C^11^), 10.0 (C^8^). **HRES-TOF** (CH_2_Cl_2_): calculated exact mass for C_18_H_33_N^120^Sn = 384.1713; measured = 384.1715: **ES-TOF** *m*/*z* = 326.0 ([M-C_4_H_9_]^+^, 52 %), 268.0 ([M-C_8_H_19_]^+^, 54 %), 211.9 ([M-C_12_H_26_]^+^, 100 %), 177.0 ([M-C_14_H_24_]^+^, 14 %), 120.9 ([M-C_18_H_32_]^+^, 37 %), 93.1 ([M-C_12_H_26_Sn]^+^, 25 %).

### Synthesis of 6, 6”-Dimethyl-2, 2’:6’,2”-terpyridine

A solution of 2, 6-dibromopyridine (1.39 g, 5.9 mmol), 2-tributylstannyl-6-methyl-pyridine (5.02 g, 13.1 mmol) and tetrakis(triphenylphosphine)palladium(0) (0.38 g, 0.33 mmol) was refluxed in degassed toluene (40 mL) for 5 days under a nitrogen atmosphere. Extra tetrakis(triphenylphosphine)palladium(0) (0.38 mg, 0.33 mmol) was added and the reaction was refluxed for a further day under a nitrogen atmosphere. The crude mixture was then concentrated in vacuo, and dichloromethane (50 mL) and 6 m hydrochloric acid (10 mL) added to form a dark brown slurry. The aqueous layer was washed with dichloromethane (2 × 50 mL) and the combined organic layers were washed with 6 m hydrochloric acid (3 × 10 mL). The combined aqueous layers were filtered and cooled in ice. Ammonia was slowly added until a light brown solid precipitated out. The resulting solid was filtered, air dried, redissolved in dichloromethane, dried with Na_2_SO_4_, and concentrated in vacuo. The crude product was purified over chromatography column (Al_2_O_3_, hexane/DCM gradient) to afford the pure 6, 6”-dimethyl-2, 2’:6’,2”-terpyridine as a white solid (0.63 g, 41 %). **^1^H NMR** (300 MHz, CDCl_3_): 8.46 (d, ^3^*J*_H3–H4_ = 7.8 Hz, 2 H, H^3^), 8.41 (d, ^3^*J*_H3a–H4a_ = 7.8 Hz, 2 H, H^3a^), 7.93 (t, ^3^*J*_H4–H3_ = 7.8 Hz, 1 H, H^4^), 7.73 (t, ^3^*J*_H4a–H3a_ ∼ ^3^*J*_H4a–H5a_ = 7.7 Hz, 2 H, H^4a^), 7.19 (d, ^3^*J*_H5a–H4a_ = 7.6 Hz, 2 H, H^5a^), 2.65 (s, 6 H, H^7^). **^13^C NMR ^1^H decoupled** (100 MHz, CDCl_3_): 158.0 (C^6a^), 155.9 (C^2/2a^), 155.8 (C^2/2a^), 137.8 (C^4^), 137.1 (C^4a^), 123.3 (C^5a^), 121.0 (C^3^), 118.3 (C^3a^), 24.8 (C^7^); **HRES-TOF** (CH_2_Cl_2_): calculated mass for C_17_H_15_N_3_Na = 284.1164; measured = 284.1152 : **ES-TOF** *m*/*z* = 284.0 [M + Na]^+^ (100 %).

### Synthesis of 6, 6”-Dibromomethyl-2, 2’:6’,2”-terpyridine

A solution of 6, 6’-dimethyl-2, 2’:6’,2”-terpyridine (0.44 g, 1.7 mmol), N-bromosuccinimide (0.77 g, 4.3 mmol) and azobisisobutyronitrile (10 mg, 0.06 mmol) in dichloromethane (30 mL) was refluxed using a 500 W halogen lamp. Progress of the reaction was monitored by TLC (SiO_2_, eluent: CH_2_Cl_2_/CH_3_OH (9/1)). After 15 hours, further N-bromosuccinimide (0.3 g, 1.7 mmol) was added and the reaction refluxed for a further 17 hours. The mixture was extracted with 0.1 m NaHCO_3_ (5 × 50 mL), the organic layers dried with Na_2_SO_4_ and concentrated in vacuo. Addition of a 50/50 mix CHCl_3_/CH_3_OH (15 mL) and storage at –20 °C overnight resulted in the formation of a white precipitate. This was collected by filtration, air-dried, and recrystallized from CHCl_3_ to afford white crystals (0.41 g, 58 %). **^1^H NMR** (300 MHz, CDCl_3_): 8.53 (d, ^3^*J*_H3–H4_ = 7.8 Hz, 2 H, H^3^), 8.52 (d, ^3^*J*_H3a–H4a_ = 7.8 Hz, 2 H, H^3a^), 7.96 (t, ^3^*J*_H4–H3_ = 7.8 Hz, 1 H, H^4^), 7.86 (t, ^3^*J*_H4a–H3a_ ∼ ^3^*J*_H4a–H5a_ = 7.8 Hz, 2 H, H^4a^), 7.50 (d, ^3^*J*_H5a–H4a_ = 7.7 Hz, 2 H, H^5a^), 4.66 (s, 4 H, H^7^). **^13^C NMR ^1^H decoupled** (100 MHz, CDCl_3_): 156.4 (C^2/2a^), 156.1 (C^2/2a^), 155.1 (C^6a^), 138.0 (C^4^), 137.0 (C^4a^), 123.6 (C^5a^), 121.6 (C^3a^), 120.4 (C^3^), 34.3 (C^7^); **HRES-TOF** (CH_2_Cl_2_): calculated exact mass for C_17_H_13_N_3_^79^Br^81^BrNa = 441.9353; measured = 441.9355 ([M + Na]^+^).

### Synthesis of bipy-GS_2_: 5, 5’-Bis(methyl-S-glutathionyl)-2, 2’-bipyridine

A solution of L-glutathione (30.3 mM, 5 mL, 0.152 mmol) in 100 mM Tris.HCl buffer pH 8.0 was added to a solution of 5, 5’-bis(bromomethyl)-2, 2’-bipyridine (15.3 mM, 76.6 μmol, 5 mL) in acetonitrile. The resulting suspension was degassed with N_2(g)_ for 10 minutes and then stirred for 11 hours at room temperature. The solvent was evaporated in vacuo at 50 °C to yield a pink gel. Deionised water (ca. 5 mL) and few drops of HCl (35 %) were added to the gel resulting in complete solubilisation. The pH was neutralised on addition of NaOH (1 m) solution. The product was purified by preparative RP-HPLC (C18 Phenomenex, monitoring absorbance at 210 and 290 nm) using 0 to 15 % gradient acetonitrile in water (containing 0.05 % TFA) over 30 min. The solvent was evaporated in vacuo to yield pure 5, 5’-bis(methyl-S-glutathionyl)-2, 2’-bipyridine as a pink solid (42 mg, 68 %). **^1^H NMR** (300 MHz, D_2_O pD ∼ 1): 8.83 (s, 2 H, H^6^), 8.42 (s, 4 H, H^3^ and H^4^), 4.53 (dd, ^3^*J*_H9–H8a_ = 5.3, ^3^*J*_H9–H8b_ = 8.4 Hz, 2 H, H^9^), 4.02 (s, 4 H, H^7^), 4.00 (m, 2 H, H^16^), 3.99 (s, 4 H, H^11^), 3.04 (dd, ^2^*J*_H8a–H8b_ = 14.2, ^3^J_H8a–H9_ = 5.4 Hz, 2 H, H^8a^), 2.86 (dd, ^2^*J*_H8b–H8a_ = 14.2, ^3^*J*_H8b–H9_ = 8.5 Hz, 2 H, H^8b^), 2.56 (m, 4 H, H^14^), 2.19 (m, 4 H, H^15^). **^13^C NMR ^1^H decoupled** (100 MHz, D_2_O): 175.0 (C^13^), 173.5 (C^12^), 173.1 (C^10^), 172.7 (C^17^), 146.6 (C^6^), 144.0 (C^4^), 139.4 (C^5^), 124.3 (C^3^), 53.4 (C^9^), 53.2 (C^16^), 41.7 (C^11^), 33.1 (C^8^), 32.8 (C^7^), 31.6 (C^14^), 26.2 (C^15^); **HRES-TOF** (water): calculated exact mass for C_32_H_43_N_8_O_12_S_2_ = 795.2442; measured = 795.2476; **ES-TOF:** *m*/*z* = 795.5 ([M + H]^+^, 100 %), 817.2 ([M + Na]^+^, 45 %).

### Synthesis of terpy-GS_2_ :6, 6”-Bis(methyl-S-glutathionyl)-2, 2’:6’,2”-terpyridine

To a suspension of 6, 6”-bis(bromomethyl)-2, 2’:6’,2”-terpyridine (2.52 mM, 25.2 μmol, 10 mL) in acetonitrile, was added 100 mM aqueous Tris.HCl buffer pH 8.0 (5 mL) and a solution of L-glutathione (20.1 mM, 5 mL, 0.100 mmol) in the same buffer. The suspension was degassed with N_2(g)_ for 10 min, heated to 45 °C and stirred at this temperature for 8 hours. The solvent was evaporated in vacuo at 50 °C to yield a colourless gel. Deionised water (ca. 5 mL) and few drops of HCl (35 % w) were added to the gel resulting in complete solubilisation. The pH was neutralised on addition of few drops of NaOH (1 m) solution. The product was purified by preparative RP-HPLC (C18 Phenomenex, monitoring absorbance at 210 nm) using 0 to 30 % gradient acetonitrile in water (containing 0.05 % TFA) over 25 min. The solvent was evaporated in vacuo to yield pure 6, 6”-bis(methyl-S-glutathionyl)-2, 2’:6’,2”-terpyridine as a white solid (17 mg, 76 %). **^1^H NMR** (300 MHz, D_2_O pD ∼ 1): 8.68 (dd, ^3^*J*_H4a–H3a_ = 8.1, ^3^*J*_H4a–H5a_ = 6.2 Hz, 2 H, H^4a^), 8.67 (dd, ^3^*J*_H3a–H4a_ = 8.1, ^4^*J*_H3a–H5a_ = 2.7 Hz, 2 H, H^3a^), 8.59 (d, ^3^*J*_H3–H4_ = 7.5 Hz, 1 H, H^3^), 8.47 (dd, ^3^*J*_H4–H3_ = 7.1, ^3^*J*_H4–H3_ = 8.8 Hz, 1 H, H^4^), 8.17 (dd, ^3^*J*_H5a–H4a_ = 6.2, ^4^*J*_H5a–H3a_ = 2.5 Hz, 2 H, H^5a^), 4.56 (dd, ^3^*J*_H9–H8b_ = 8.2, ^3^*J*_H9–H8a_ = 5.5 Hz, 2 H, H^9^), 4.38 (s, 4 H, H^7^), 4.01 (t, ^3^*J*_H16–H15_ = 6.6 Hz, 2 H, H^16^), 3.89 (s, 4 H, H^13^), 3.15 (dd, ^2^*J*_H8a–H8b_ = 14.2, ^3^*J*_H8a–H9_ = 5.6 Hz, 2 H, H^8a^), 2.94 (dd, ^2^*J*_H8b–H8a_ = 14.2, ^3^*J*_H8b–H9_ = 8.3 Hz, 2 H, H^8b^), 2.51 (dd, ^3^*J*_H14–H15a_ = 7.0, ^3^*J*_H14–H15b_ = 7.9 Hz, 4 H, H^14^), 2.14 (m, 4 H, H^15^). **^13^C NMR ^1^H decoupled** (125 MHz, D_2_O): 174.8 (C^13^), 173.3 (C^12^), 172.7 (C^10^), 172.1 (C^17^), 155.8 (C^6a^), 148.4 (C^2/2a^), 148.1 (C^2/2a^), 147.7 (C^4a^), 142.5 (C^4^), 128.6 (C^5a^), 126.2 (C^3^), 124.3 (C^3a^), 53.3 (C^9^), 52.8 (C^16^), 41.6 (C^11^), 33.9 (C^8^), 33.6 (C^7^), 31.5 (C^14^), 26.0 (C^15^); **HRES^–^-TOF** (water): calculated exact mass for C_37_H_45_N_9_O_12_S_2_Na = 894.2527; measured = 894.2498; **ES-TOF:** *m*/*z* = 894.7 ([M + Na]^+^, 100 %), 872.7 ([M + H]^+^, 40 %), 589.5 ([M-C_10_H_16_N_3_O_6_S+Na]^+^, 17 %), 567.5 ([M-C_10_H_16_N_3_O_6_S+H]^+^, 19 %).

### Circular Dichroism (CD) Spectroscopy

CD spectra were recorded in 1 mm pathlength quartz cuvettes at 298 K with a Jasco J-715 spectropolarimeter. The observed ellipticities in millidegrees were converted into molar ellipticity, *Φ*, and are reported in units of deg dmol^–1^ cm^2^. Metal-titrations of model compounds were performed by addition of aliquots of a 21 mM stock solution of CuCl_2_ or ZnCl_2_, into a 350 μM solution of **bipy-GS_2_**/**terpy-GS_2_** in 10 mM phosphate buffer pH 7.4. Spectra did not change over two minutes, and therefore samples were allowed to equilibrate for two minutes prior to measurement. Reported spectra are an average of 5 scans recorded between 200 and 400 nm at 200 nm/min (0.5 nm pitch) and the buffer blank subsequently subtracted (except for Cu titration of **terpy-GS_2_**). After two (Zn) or three (Cu) equivalents of metal-ion were added, 21 or 31.5 μL respectively of a 0.2 m EDTA stock solution was added to assess reversibility (20 equivalents of EDTA per metal ion).

### UV/Vis Spectroscopy

UV/Vis spectra were recorded in a 1 cm pathlength quartz cuvette at 298 K with a Shimadzu 1800 spectrometer. For the pH titration of model switches, aliquots of HCl, NaOH (**terpy-GS_2_**) or KOH (**bipy-GS_2_**) solutions of various concentrations (0.01, 0.1, 1 m) were added to cuvettes containing 3 mL of a 5 μM solution of the model switch. The solution was allowed to equilibrate for 10 min prior to recording the pH on a Jenway 3510 pH meter, and recording a UV/Vis spectrum.

For the metal titrations, aliquots of an aqueous 0.75 mM stock solution of either CuCl_2_ or ZnCl_2_, were titrated into 3 mL of a 5 μM solution of model switches in 20 mM potassium phosphate buffer pH 7.4, and the spectra recorded after 3 min equilibration. Non-linear fitting were performed with Kaleidagraph software version 4.0. K_app_ values were calculated by fitting data for the absorbance maximum of the metal complexes as a function of Cu^II^/Zn^II^ concentration, to Equation (1) and (2):

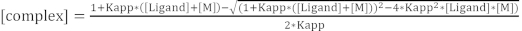
(1)

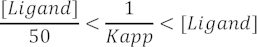
(2)

The constant b corresponds to the cuvette pathlength, [complex] corresponds to the concentration of 1:1 complex, [Ligand] the total **bipy-GS_2_** or **terpy-GS_2_** and [M] the total CuCl_2_/ZnCl_2_ concentration at each point.

In order to ensure an accurate estimation, measurements were performed at concentrations close to the apparent dissociation constants, such that:

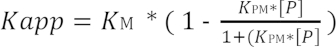


The *K_M_* values were corrected by accounting for the contribution from phosphate metal ion binding, based on values reported in the literature,^21^ see Equation (3):

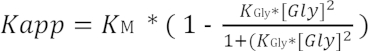
(3)

This is based on the assumption that the amount of free phosphate is equal to the total amount of phosphate in solution. *K_M_* and *K_PM_* corresponds to the estimated binding constant of the metal ion to the ligand of interest and the phosphate anion, respectively. When glycine was added as a competitor, *K_M_* was estimated using Equation (4), based on the reported Cu^II^ binding constant for glycine.^47^ The contribution from the phosphate anion was regarded as negligible, when 20 mM glycine was present.

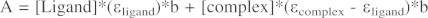
(4)

### NMR Spectroscopy

All ^1^H and ^13^C NMR spectra were collected with either Bruker DRX500 (500 MHz ^1^H and 125 MHz ^13^C, *T* = 300 K), AVIII400 (400 MHz ^1^H and 100 MHz ^13^C, *T* = 298 K) or AVIII300 (300 MHz ^1^H, *T* = 293 K) spectrometer equipped with a 5 mm probe. Titrations were performed by addition of aliquots of a 0.1 m stock solution of ZnCl_2_ in D_2_O to a 5 mM solution of polypyridyl-conjugate in 50 mM phosphate buffer pD 7.4 in D_2_O, and either 4 mM dioxane (*δ* = 3.75 ppm) or 1 mM acetone (*δ* = 2.22 ppm) was used as an internal reference. Addition of two equivalents ZnCl_2_ resulted in no more than a 10 % increase to the total volume. 0.8 mL of a 0.25 m solution of ethylediaminetetracetic acid (EDTA) in D_2_O (pH adjusted to 8), and 100 μL D_2_O, were then added (20 equivalents EDTA vs. Zn) to the NMR sample, resulting in a dilution of the sample by two. Changes in peak integrations (see Figure 6C and 66D) are reported relative to the internal dioxane reference.

2D NMR of **terpy-GS_2_** in D_2_O at acidic pD (dioxane internal reference), were first recorded on AVIII400 (400 MHz, *T* = 298 K): DQF-COSY and Gradient NOESY spectra (400 ms mixing time). Additionally, Gradient NOESY (States-TPPI, 450 ms mixing time) and Phase cycle ROESY (States-TPPI, 450 ms mixing time, 10 KHz spin lock field and an offset of 10 KHz in spin lock period to minimise HOHAHA effects) experiments were recorded on DRX500 (500 MHz, *T* = 300 K, partial presaturation of water signal), both displaying the previously mentioned inter-ring coupling.

Chemical shifts (δ) are given in parts per million (ppm) to higher frequency compared to the methyl signal of the sodium salt of 3-(trimethylsilyl)propanesulfonicacid at 0 ppm.^48^ Data were processed using Bruker Topspin version 2.1 (300 and 400 MHz) or 1.3 (500 MHz) and Mestrenova Lite version 5.2.5.

### Ion Mobility Spectrometry (IMS) Mass Spectrometry (MS)

Samples of **bipy-GS_2_/terpy-GS_2_** in methanol:water (1:1) were diluted to a final concentration of 5 μM and the pH adjusted to 6.7, prior to infusion. Similar solutions were prepared with 1.0 equivalent of either CuCl_2_ or ZnSO_4_, and 1.4 (**bipy-GS_2_**) or 1.9 equivalent (**terpy-GS_2_**) of CuCl_2_, the pH adjusted and the samples allowed to equilibrate for 10 minutes prior to subsequent dilution and infusion. IMS was used to separate ions in the gas phase based on their mobility in the nitrogen carrier gas, and subsequently identify them by ESI-MS. IMS-MS experiments were performed on a Waters Synapt G2 based on a previously reported procedure.^49^ The T-Wave ion mobility device was calibrated with a species of known collision cross-section (CCS) determined using standard drift tube instruments. Polyalanine was employed as a calibrant to measure against and include into our collision cross-section (CCS) calculation.

**Supporting Information** (see footnote on the first page of this article): Analytical HPLC (Figure S1), UV/Vis pH titrations (Figure S2), ^1^H NMR spectra (Figures S3 – S5) and IMS-MS spectra for **bipy-GS_2_** in the absence and presence of Cu/Zn (Figure S6) are reported.

## Notes

As a service to our authors and readers, this journal provides supporting information supplied by the authors. Such materials are peer reviewed and may be re-organized for online delivery, but are not copy-edited or typeset. Technical support issues arising from supporting information (other than missing files) should be addressed to the authors.
