# Supplementary material for: Conformational Study of an Artificial Metal-Dependent Regulation Site for Use in Designer Proteins
Source: Z Anorg Allg Chem. 2013 May 21;639(8-9):1370–83. doi: 10.1002/zaac.201300131 (PMC4431501; doi:10.1002/zaac.201300131)
Supplement: Supplementary file 1 [file zaac0639-1370-sd1.pdf]

Z. Anorg. Allg. Chem. **2013** · © WILEY-VCH Verlag GmbH & Co. KGaA, 69451 Weinheim, 2013 · ISSN 0044–2313

SUPPORTING INFORMATION

Title: Conformational Study of an Artificial Metal-Dependent Regulation Site for Use in Designer Proteins

Author(s): E. Oheix, N. Spencer, L. A. Gethings, A. F. A. Peacock*

Ref. No.: Z201300131

Supporting information

Conformational study of an artificial metal-dependent regulation site for use in designer proteins

*Emmanuel Oheix, Neil Spencer, Lee A. Gethings and Anna F. A. Peacock**

E-mail: a.f.a.peacock@bham.ac.uk

Content:

Figure S1 – Analytical HPLC of model switches

Figure S2 – UV-visible pH titration of model switches

Figure S3 – Representation of the relationship between energetic profiles and ^{1}H NMR spectra for **bipy-GS$_2$**

Figure S4 - ^{1}H NMR spectra of **terpy-GS$_2$** at pD 1 and 7.4

Figure S5 - 2D NMR spectra of **terpy-GS$_2$**

Figure S6 - Ion-mobility MS spectra for **bipy-GS$_2$** in the absence and presence of Cu/Zn

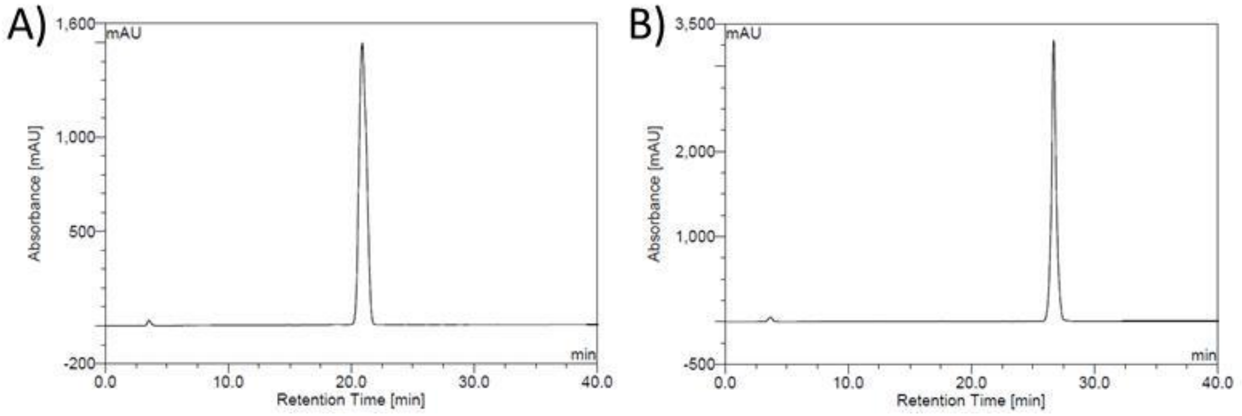

Figure S1 – Analytical reverse phase C18-HPLC chromatograph of pure **bipy-GS$_2$** and **terpy-GS$_2$** using H_2O/MeCN gradient (monitoring at 220 nm). A) **Bipy-GS$_2$** (gradient 0 – 25 % MeCN over 40 minutes) and B) **terpy-GS$_2$** (gradient 0 – 30 % MeCN over 40 minutes). Peak at R_T = 3.7 min is present in the blank.

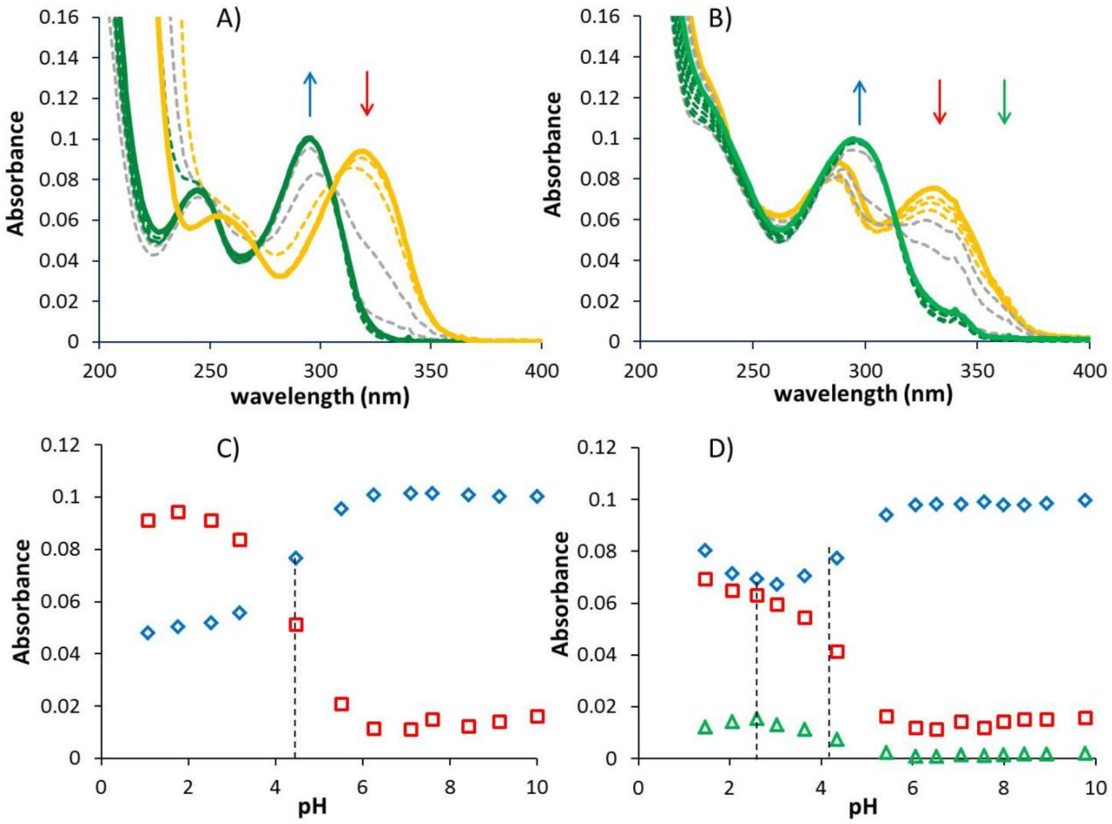

Figure S2 – pH Titration of model compounds monitored by UV-vis. (top) UV spectrum of 5 μM A) **bipy-GS$_2$** and B) **terpy-GS$_2$** recorded at pH values between 1.5 and 10, (▬) acidic, (▪▪▪) intermediary, (▬) basic. (Bottom) plot of the absorbance maxima as a function of pH for C) **bipy-GS$_2$** (295 nm, ◊; and 319 nm, □) and D) **terpy-GS$_2$** (295 nm, ◊; 340 nm, □; and 367 nm, Δ).

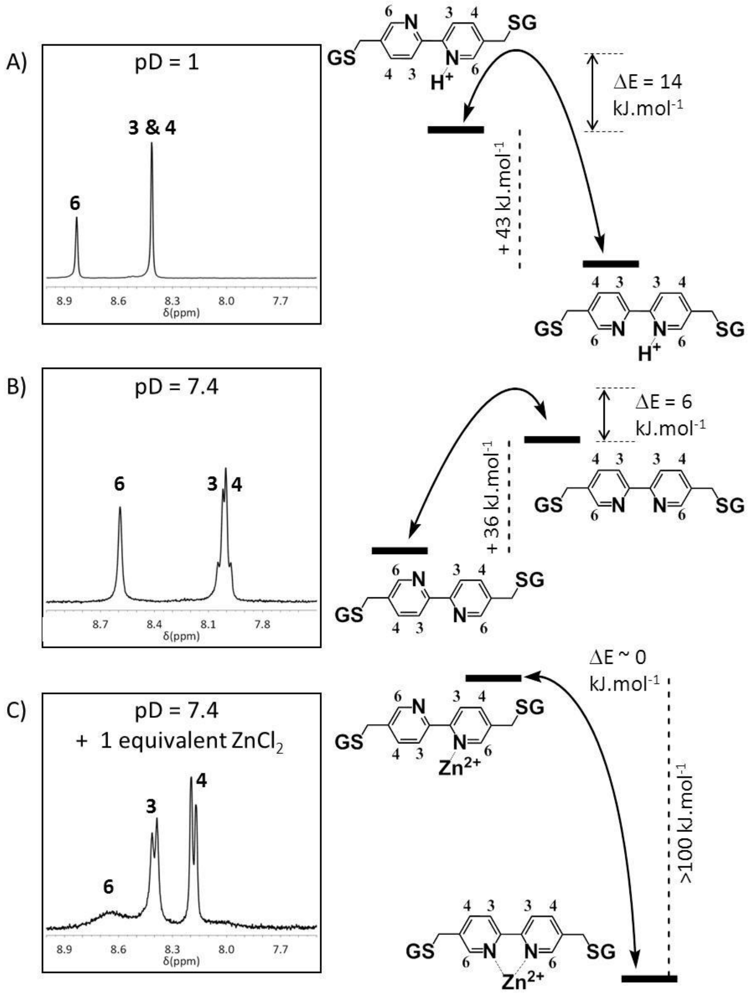

Figure S3 – Illustration of the relationship between energy profiles and ^{1}H NMR spectra for **bipy-GS$_2$**. ^{1}H NMR spectra of **bipy-GS$_2$** are more greatly influenced by conformational flexibility rather than the conformation of the lowest energy structure. Representation of energy profiles regulating the population of the different conformers of the A) monoprotonated **bipy-GS$_2$**, B) **bipy-GS$_2$** and C) when complexed to Zn(II), alongside ^{1}H NMR spectra (D_2O, 300 MHz, 293 K). Energy profiles and activation barriers are based on *ab initio* reports for the *cisoïd-transoïd* interconversion of 2,2'-bipyridine.[1-3] The impact of substitutions at position 5 and 5' on the rotation of the 2,2'-bipyridine can be ignored.[4]

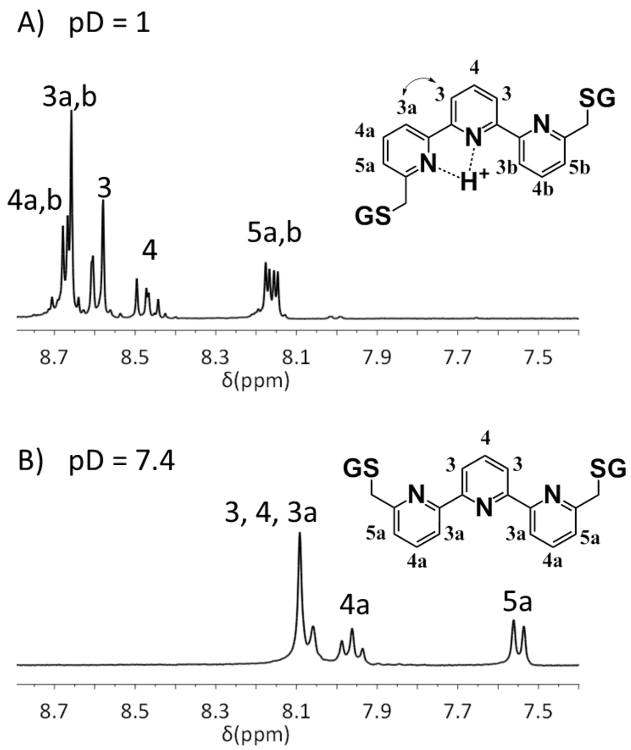

Figure S4 – ^{1}H NMR spectra of **terpy-GS$_2$** at differing pD. ^{1}H NMR spectrum of A) 9 mM **terpy-GS$_2$** recorded at pD ~1 (500 MHz, 300 K), and B) 5 mM **terpy-GS$_2$** buffered at pD 7.4 with 50 mM phosphate buffer (300 MHz, 293 K).

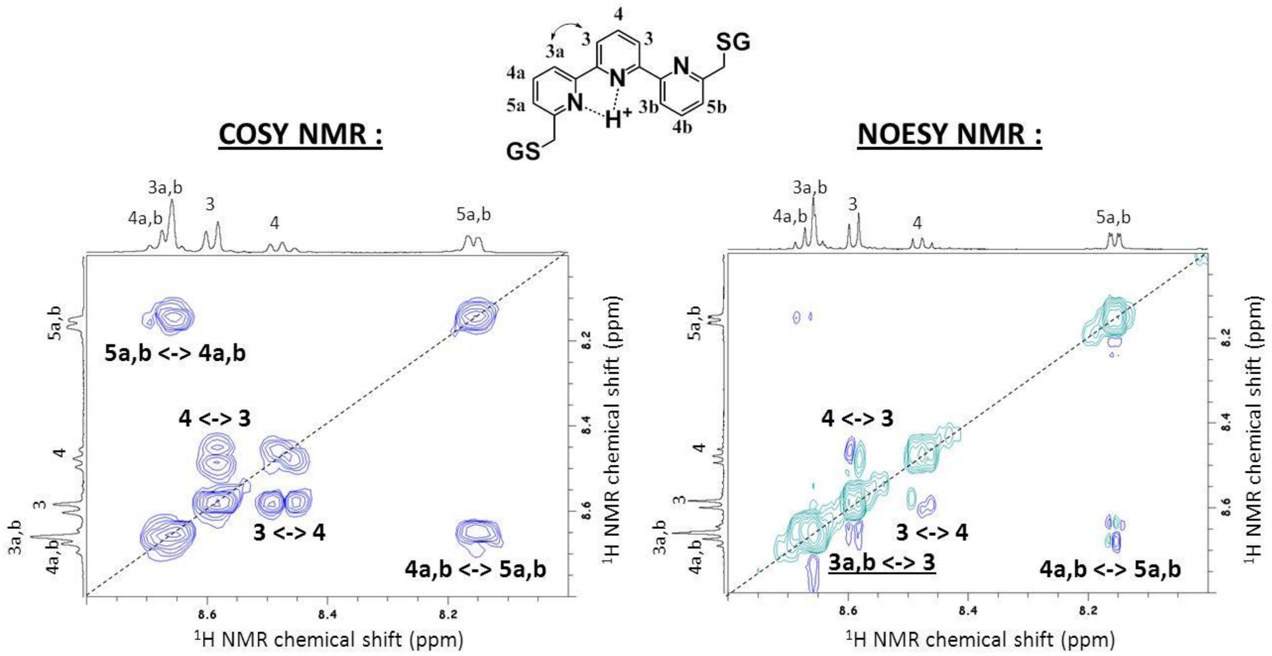

Figure S5 – 2D NMR of **terpy-GS$_2$** 9 mM solution in D_2O (pD ~1, 300 K). COSY spectrum recorded at 400 MHz; NOESY spectrum recorded at 500 MHz, 450 ms mixing time.

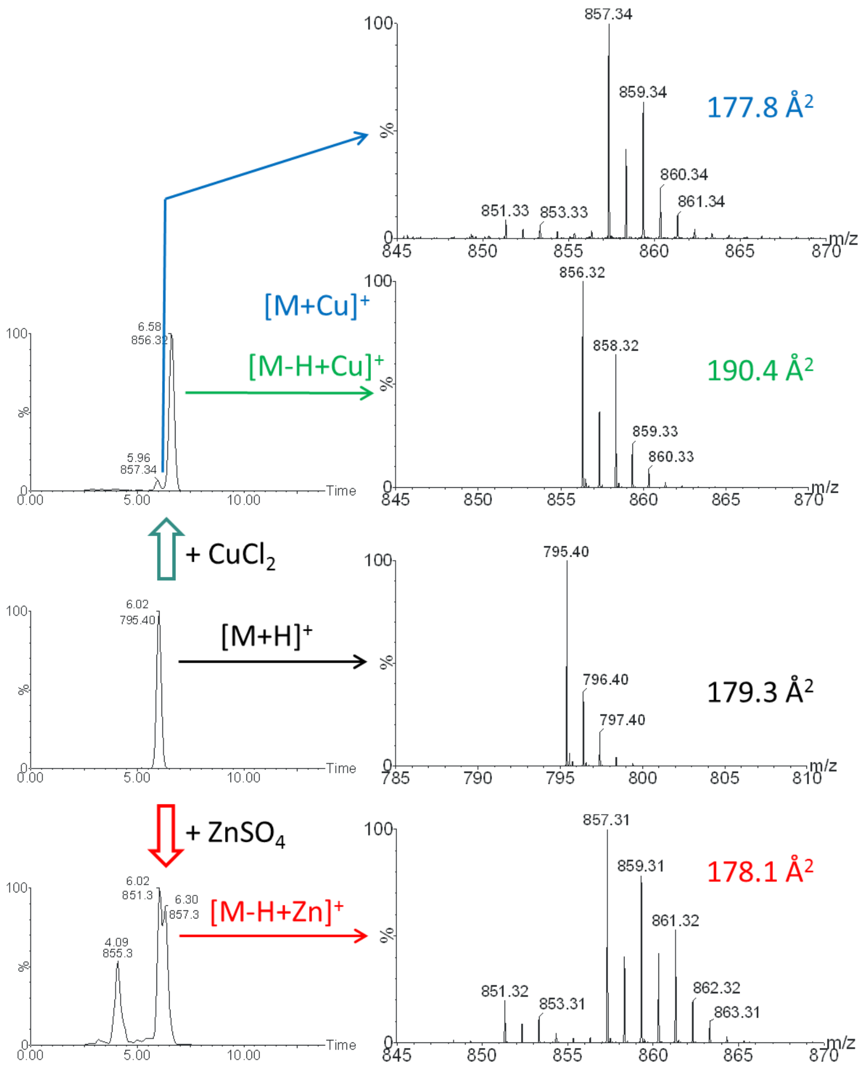

Figure S6 – Ion-mobility mass spectrometry spectra on addition of Cu(II) or Zn(II) to **bipy-GS$_2$**. Drift times (ms) for $[M+H]^+$ ion of **bipy-GS$_2$**; $[M+Cu]^+$ and $[M\text{-}H+Cu]^+$ ions of **bipy-GS$_2$** in the presence of 1 equivalence $CuCl_2$; and the $[M\text{-}H+Zn]^+$ ion of **bipy-GS$_2$** in the presence of 1 equivalence $ZnSO_4$. Associated ESI-MS spectra of these ions, and their calculated cross sections.

References

(1) Howard S. T., *J. Am. Chem. Soc.*, **1996**, 118, 10269-10274.

(2) Göller A., Grummt U., *Chem. Phys. Lett.*,**2002**, 232-242.

(3) Grummt U., Erhardt S., *J. Mol. Struc-Theochem.*, **2004**, 685, 133-137.

(4) Zahn S., Reckien W., Kirchner B., Staats H., Matthey J., Lützen A., *Chem. Eur. J.*, **2009**, 2572-2580.
